# Dissecting nematode resistance regions in soybean revealed pleiotropic effect of soybean cyst and reniform nematode resistance genes

**DOI:** 10.1002/tpg2.20083

**Published:** 2021-03-16

**Authors:** Mariola Usovsky, Naoufal Lakhssassi, Gunvant B. Patil, Tri D. Vuong, Sarbottam Piya, Tarek Hewezi, Robert T. Robbins, Robert M. Stupar, Khalid Meksem, Henry T. Nguyen

**Affiliations:** ^1^ Division of Plant Sciences University of Missouri Columbia MO USA; ^2^ Department of Plant Soil and Agricultural Systems Southern Illinois University Carbondale IL USA; ^3^ Institute of Genomics for Crop Abiotic Stress Tolerance Department of Plant and Soil Science Texas Tech University Lubbock TX USA; ^4^ Department of Plant Sciences University of Tennessee Knoxville TN USA; ^5^ Department of Plant Pathology University of Arkansas Fayetteville AR USA; ^6^ Department of Agronomy and Plant Genetics University of Minnesota St. Paul MN USA

## Abstract

Reniform nematode (RN, *Rotylenchulus reniformis* Linford & Oliveira) has emerged as one of the most important plant parasitic nematodes of soybean [*Glycine max* (L.) Merr.]. Planting resistant varieties is the most effective strategy for nematode management. The objective of this study was to identify quantitative trait loci (QTL) for RN resistance in an exotic soybean line, PI 438489B, using two linkage maps constructed from the Universal Soybean Linkage Panel (USLP 1.0) and next‐generation whole‐genome resequencing (WGRS) technology. Two QTL controlling RN resistance were identified—the soybean cyst nematode (SCN, *Heterodera glycines*) resistance gene *GmSNAP18* at the *rhg1* locus and its paralog *GmSNAP11*. Strong association between resistant phenotype and haplotypes of the *GmSNAP11* and *GmSNAP18* was observed. The results indicated that *GmSNAP11* possibly could have epistatic effect on *GmSNAP18*, or vice versa, with the presence of a significant correlation in RN resistance of *rhg1‐a GmSNAP18* vs. *rhg1‐b GmSNAP18*. Most importantly, our preliminary data suggested that GmSNAP18 and GmSNAP11 proteins physically interact *in planta*, suggesting that they belong to the same pathway for resistance. Unlike *GmSNAP18*, no indication of *GmSNAP11* copy number variation was found. Moreover, gene‐based single nucleotide polymorphism (SNP) markers were developed for rapid detection of RN or SCN resistance at these loci. Our analysis substantiates synergic interaction between *GmSNAP11* and *GmSNAP18* genes and confirms their roles in RN as well as SCN resistance. These results could contribute to a better understanding of evolution and subfunctionalization of genes conferring resistance to multiple nematode species and provide a framework for further investigations.

## Core Ideas

- RN resistance was mapped to regions containing *rhg1* (GmSNAP18), and its paralog (GmSNAP11).
- *GmSNAP18* and *GmSNAP11* exhibit pleiotropic effect on resistance to RN and SCN.
- The combination of *rhg1‐a* GmSNAP18 and GmSNAP11 provides high resistance to RN and SCN.
- GmSNAP18 and GmSNAP11 proteins physically interact in planta.

**Abbreviations**
BiFC: bimolecular fluorescence complementation
CGH: comparative genomic hybridization
Chr.: chromosome
CNV: copy number variation
KASP: Kompetitive allele specific PCR
LG: linkage group
LOD: logarithm of the odds
MG: maturity group
MQM: multiple‐QTL modeling
NAM: nested association mapping
QTL: quantitative trait loci
RI: reproduction index
RIL: recombinant inbred line
RKN: root‐knot nematode
RN: reniform nematode
SCN: soybean cyst nematode
SNAP: soluble N‐ethylmaleimide‐sensitive factor (NSF) attachment protein
SNP: single nucleotide polymorphism
SSR: simple sequence repeat
TPR: tetratricopeptide repeat
WGRS: whole‐genome resequencing
YFP: yellow fluorescent protein

## 1 INTRODUCTION

Reniform nematode (RN, *Rotylenchulus reniformis* Linford & Oliveira), a microscopic root parasite, is the most economically important species among the *Rotylenchulus* genus that causes plant stunting, yellowing of the foliage, and yield reduction as a direct result of root damage (Allen et al., 2017; Cook, Robinson, & Namken, 1997). Among crop plants, most severely affected by RN are upland cottonb (*Gossypium hirsutum* L.), cowpea [*Vigna unguiculata* (L.) Walp.], soybean [*Glycine max* (L.) Merr.], pineapple [*Ananas comosus* (L.) Merr. var. *comosus*], and many vegetable crops including tomato (*Solanum lycopersicum* L.), okra [*Abelmoschus esculentus* (L.) Moench], squash (*Cucurbita maxima* Duchesne), and lettuce (*Lactuca sativa* L.) (Cook et al., 1997). This nematode is found throughout the southern United States and, unlike other nematode species, it can survive and reproduce in nearly any soil type (Koenning, Walters, & Barker, 1996). Crop rotation with resistant or immune plant species is recommended as a field management option (Davis, Koenning, Kemerait, Cummings, & Shurley, 2003; Stetina, Smith, & Ray, 2014).

Numerous soybean germplasm, breeding lines, and cultivars have been screened over many years for RN resistance (Robbins & Rakes, 1996; Robbins, Rakes, & Elkins, 1994, 1999). Unfortunately, there are very few soybean cultivars in the maturity group (MG) IV–VII range with high level of RN resistance. Soybean cultivars ‘Peking’ (MG IV), ‘Dyer’ (MG V), ‘Custer’ (MG IV), ‘Pickett’ (MG VI), ‘Forrest’ (MG V), ‘Hartwig’ (MG V), and ‘Anand’ (MG V) are commonly reported as resistant to RN (Davis, Koenning, Burton, & Barker, 1996; Robbins et al., 1994, Rebois, Johnson, & Cairns, 1968). Moreover, limited genetic mapping work was performed in the past. Ha et al. (2007) mapped one major RN resistance quantitative trait loci (QTL) to chromosome (Chr.) 19 (linkage group [LG] L) and two minor QTL on Chrs. 11 (LG B1) and 18 (LG G) in PI 437654. Jiao et al. (2015) mapped two minor RN resistance QTL on Chrs. 11 and 18 in PI 567516C. Recently, Lee, Lightfoot, Anderson, Robbins, and Kantartzi (2016) mapped two minor RN QTL on Chrs. 12 (LG H) and 18 (LG G) in cultivar Hartwig, which was derived from PI 437654 and Forrest. The RN resistance genes within mapped QTL are still elusive because of a lack of high‐resolution maps and, as a result, reliable diagnostic single nucleotide polymorphism (SNP) markers are not available to support marker‐assisted selection.

For many years, nematode research in soybean primarily focused on soybean cyst nematode (SCN, *Heterodera glycines*) as the main concern of soybean yield losses. Much less effort has been done for other nematode species, such as RN and root‐knot nematode (RKN, *Meloidogyne incognita*). These two species, however, also cause severe damage, especially in the southern and southeastern states, requiring a strong need for additional research. More attention to RN host resistance is of high importance to isolate the genes and to study their molecular mechanisms. Searching for new sources of resistance and their introduction into high‐yielding backgrounds, in combination with other nematode species, is essential to provide broad‐based and durable resistance against multi‐nematode populations (Brzostowski & Diers, 2017; Robinson et al., 2007). The objective of this research was to map genomic region conferring RN resistance in a soybean accession PI 438489B using the Universal Soybean Linkage Panel (USLP 1.0) and next‐generation whole‐genome re‐sequencing (WGRS) technology. The results pinpointed two *GmSNAP* paralogs that were closely analyzed using evolutionary and structural analyses.

**Core Ideas**

- RN resistance was mapped to regions containing *rhg1* (GmSNAP18), and its paralog (GmSNAP11).
- *GmSNAP18* and *GmSNAP11* exhibit pleiotropic effect on resistance to RN and SCN.
- The combination of *rhg1‐a* GmSNAP18 and GmSNAP11 provides high resistance to RN and SCN.
- GmSNAP18 and GmSNAP11 proteins physically interact in planta.

## 2 MATERIALS AND METHODS

### 2.1 Plant materials and RN bioassay

A population of 247 F_8_ recombinant inbred lines (RILs), derived from a ‘Magellan’ × PI 438489B cross (Vuong, Sleper, Shannon, Wu, & Nguyen, 2011), and the parental lines were evaluated for resistance to RN in a greenhouse at the University of Arkansas, Fayetteville, AR, following a well‐established protocol as previously described (Robbins, Rakes, Jackson, & Dombek, 1999). The reproduction index (RI) was calculated as the number of nematodes at test termination (Pf)/initial infestation density (Pi). The RI value was scored based on the average of five technical replicates for each RIL and used for statistical analysis and QTL mapping. The frequency distribution of RI values among 247 RILs, the normality test using Shapiro–Wilk (*w*) and skewness statistics were performed using SAS software version 9.1 (SAS Institute Inc.).

### 2.2 QTL mapping

To map QTL for RN resistance in PI 438489B, two separate genetic analyses were conducted using linkage maps constructed from the USLP 1.0 array and WGRS technology. The RIL population was genotyped using USLP 1.0 (Hyten et al., 2010) and simple sequence repeat (SSR) markers (Vuong et al., 2011). A total of 700 SNP and 204 SSR polymorphic markers were used for linkage map construction (Vuong et al., 2011). For WGRS analysis, the multiplexed Illumina sequencing standard protocol was used for 247 RILs, with effective sequence depth range between 0.10× to 0.31× with an average of 0.19×. The corresponding sequence coverage ranged from 8.84 to 24.75% with a mean of 16.2% (Xu et al., 2013). Bin map was used as a molecular marker data to construct a linkage map using the software R/qtl package (Broman, 2009; Xu et al., 2013). The genetic linkage maps were constructed using JoinMap 4.0 (van Ooijen, 2006). The logarithm of the odds (LOD) threshold score of 3.0 and a maximum genetic distance of 50 cM were used for the initial linkage grouping of markers. The soybean genetic linkage groups were assigned with the markers corresponding to Chr. numbers.

Mapping of QTL was performed using MapQTL 5.0 software (van Ooijen, Boer, Jansen, & Maliepaard, 2000). Permutation tests were conducted in analyzed RILs for 1,000 times, and initial LOD threshold was 3.0 under type I error 0.05. Interval mapping at 1‐cM intervals along the chromosomes was used to detect QTL based on LOD threshold of 3.0. Markers closely linked to positions with the highest LOD scores were taken as cofactors for multiple‐QTL modeling (MQM) analysis (van Ooijen et al., 2000). Selection of candidate genes for each detected QTL interval was annotated in Glyma.Wm82.a2.v1 assembly using the SoyBase database (www.soybase.org). Further prediction was referred to the genes of known function in soybeans related to plant resistance.

### 2.3 Sequence‐similarity analysis

To understand the sequence‐similarity relationship, SNP calling of 107 sequenced soybean accessions (Valliyodan et al., 2016) were used to construct trees based on two mapped physical regions Gm11:32892556‐32978390 and Gm18:1502699‐1778708. The SNP data were converted to Hapmap (.hmp) format and exported as phylip interleaved using TASSEL software (Bradbury et al., 2007). The neighbor‐joining model implemented in the MEGA6 (Tamura, Stecher, Peterson, Filipski, & Kumar, 2013; MEGA Inc.), as described previously (Patil et al., 2016), was used to construct sequence similarity trees. The numbers at the nodes represent bootstrap percentage value based on 1,000 replications.

### 2.4 Haplotype analysis and copy number variation of *GmSNAPs*

The WGRS data (Valliyodan et al., 2016) of 107 diverse lines, sequenced in 15× depth, were used to identify sequence variations (SNPs, InDels) within the candidate genes. Haplotypes of the two genes, *GmSNAP11* and *GmSNAP18*, were investigated by generating map and genotype data files using TASSEL 5.0 software (Bradbury et al., 2007) and clustering pictorial output for QTL, additionally, the candidate gene region was visualized using FLAPJACK (Patil et al., 2016). Transcript sequence‐based annotation was used to classify synonymous and nonsynonymous SNPs, and prediction of the SNPs effect was performed using SnpEff (http://snpeff.sourceforge.net). Correlation between copy number variation (CNV) of *GmSNAP11* and *GmSNAP18* was generated based on comparative genomic hybridizations (CGHs) as described by McHale et al. (2012). The nested association mapping (NAM) population data for CGH and WGRS were accessed from the Stupar Laboratory, University of Minnesota, MN (http://stuparlabcnv.cfans.umn.edu:8080/).

### 2.5 Structural differences and relationship between *SNAP* genes

Structural differences between genes *GmSNAP11* and *GmSNAP18* were analyzed using clustering of SNP calls as well as amino acid sequences. To test relationship dependency between *GmSNAP11* and *GmSNAP18*, 12 RILs of the ‘Essex’ (susceptable) × Forrest (resistant) population with various combinations with the presence or absence of *GmSNAP11* and *GmSNAP18* genes were selected for RN phenotyping according to the protocol of the University of Arkansas (Robbins et al., 1999).

### 2.6 Marker development

Haplotype analysis was used to develop Kompetitive allele specific polymerase chain reaction (KASP) assay. Two SNPs, Gm11:32968127 (SNAP11‐1; 3′ untranslated region) and Gm11:32970557 (SNAP11‐2; fifth intron), were used to develop KASP assays of the *GmSNAP11* gene. Also, two SNPs, Gm18:1645012 (SNAP18‐1; eighth intron) and Gm18:1643107 (SNAP18‐2; fifth intron), were selected for the development of KASP assays of the *GmSNAP18* gene. Two previously reported markers for SCN testing of SNAP18, *Rhg1‐2* and *Rhg1‐5* (Kadam et al., 2016), were also added to compare the results. The reaction mixture was prepared according to the standard protocol described by LGC Genomics, LLC (http://www.lgcgroup.com). The fluorescent end‐point genotyping method was carried out using Roche LightCycler 480‐II instrument (Roche Applied Sciences). DNA was extracted using a nonhazardous method (King, Serrano, Boerma, & Li, 2014).

### 2.7 Protein homology modeling

Homology modeling of a putative GmSNAP18 protein structure was conducted as described earlier (Liu et al., 2017). Homology modeling of a putative GmSNAP11 and GmSNAP11‐T1 (nonsense mutations in gene *GmSNAP11* at position A240* resulted in a truncated protein) protein structures were conducted employing Deepview and Swiss Model Workspace software using GmSNAP11 and GmSNAP_A240*_ protein sequences from Forrest (Lakhssassi et al., 2017b), and an α‐SNAP crystal structure from *Rattus norvegicus* (as a template); (3J96 chain G) (Arnold, Bordoli, Kopp, & Schwede, 2006; Guex & Peitsch, 1997; Schwede, Kopp, Guex, & Peitsch, 2003; Zhao et al., 2015). Residues from 6 to 284 were modeled against this template with a sequence identity of 39%. Tetratricopeptide repeat (TPR) domains and haplotype mapping and visualizations were performed using the University of California, San Francisco (UCSF) Chimera package (Pettersen et al., 2004). After building the homology models, interaction analysis of both protein structures were tested using the GRAMM‐X protein‐docking server (Tovchigrechko & Vakser, 2005), as previously described by Lakhssassi et al. (2020a, 2020b). Mapping and visualizations of naturally occurring mutations at the GmSANP18 and GmSNAP11 proteins were performed using the UCSF Chimera package as shown earlier (Lakhssassi et al., 2017a).

### 2.8 Bimolecular fluorescence complementation assay

Coding sequences of Forrest and Essex *GmSNAP11* and GmSNAP_A240*_ wild‐type were cloned into *pSAT4‐nEYFP‐C1* to generate nEYFP‐SNAP11 and nEYFP‐SNAP_A240*_ fusions (Supplemental Figures S2–S4). Likewise, *GmSNAP18* from Forrest and Essex were cloned into *pSAT4‐cEYFP‐C1‐B* to generate cEYFP‐GmSNAP18 fusions (Supplemental Figures S5 and S6). Various combinations of *cEYFP* and *nEYFP* fusions including controls were coexpressed in onion epidermal cells by particle bombardment as described earlier (Lakhssassi et al., 2019, 2020a). Onion tissues cotransformed with *cEYFP* and *nEYFP* fusions were incubated in the dark at 25 °C. After 36 h, the tissue was examined for yellow fluorescent protein (YFP) activity. Fluorescent and bright field images were captured using EVOS FL Auto Cell Imaging System (Life Technologies). The bimolecular fluorescence complementation technology used in the current study was successfully employed recently to validate the presence of the multi‐protein complex involving GmSNAP18, GmSHMT08, and GmPR08‐Bet VI proteins in resistance to SCN (Lakhssassi et al., 2020b).

## 3 RESULTS

### 3.1 Phenotypic variation and QTL associated with RN resistance

**FIGURE 1:**
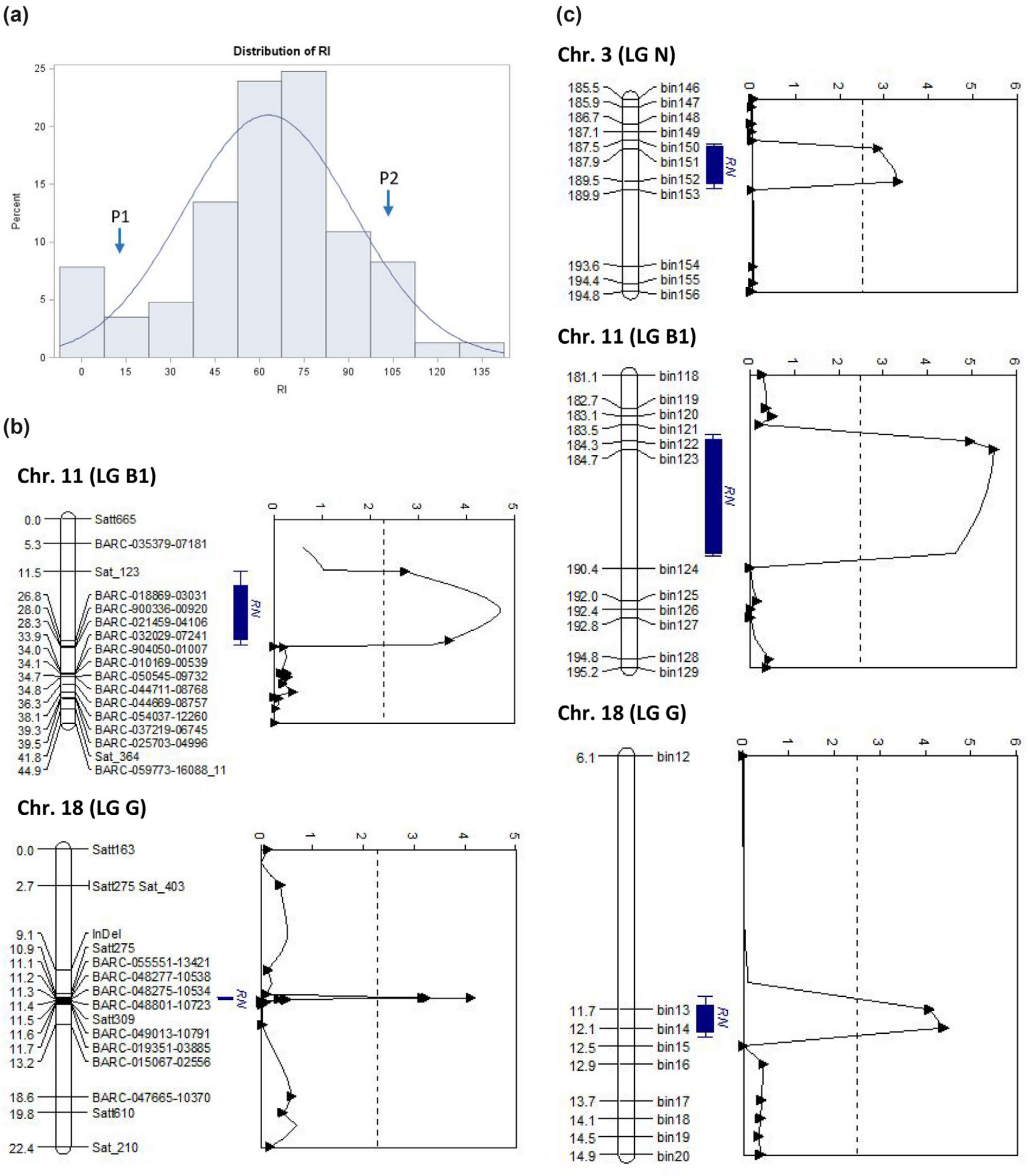
Quantitative trait loci mapping for reniform nematode resistance: (a) Frequency distribution of reproduction indices (RI) within a F_7:8_ RIL ‘Magellan’ × PI 438489B population. A lower RI value indicates a higher level of resistance. P1 and P2 arrows indicate parental lines PI 438489B and Magellan, respectively; (b) QTL mapped to chromosomes 11 and 18 using the USLP 1.0 array with a genome‐wide logarithm of the odds (LOD) threshold of 2.3; (c) QTL mapped to chromosomes 3, 11, and 18 using the WGRS (bin map) with a genome‐wide LOD threshold of 2.3

The parental lines, Magellan and PI 438489B, displayed a significant difference in RI equal to 80 and 1.5, respectively (Figure 1a; Table 1). The frequency distribution of RI values among 247 RILs showed large genetic variation, ranging between 0.6 and 137 with a mean of 63 and median of 55. The normality test by the Shapiro–Wilk (*w*) and Skewness statistics indicated that the RI in the population was not normally distributed.

**TABLE 1:** A summary of statistics of the parental lines and 247 F_7:8_ recombinant inbred lines (RILs) developed from a ‘Magellan’ × PI 438489B cross for their responses to reniform nematode. The normality tests of reniform nematode (RN) reproductive index are shown by Shapiro‐Wilk (*w*), skewness, and kurtosis. Broad‐sense heritability (*h* ^2^_b_) was estimated based on the ANOVA outputs

RN reproductive index									
Parents		247 F_7:8_ recombinant inbred lines							
Magellan	PI 438489B	Mean	Min.	Max.	SD	*w*	Skewness	Kurtosis	*h* ^2^_b_
————————————%————————————									
79.8	1.5	61.5	0.6	136.9	28.5	.97	−0.34	.07	.66

The first approach for QTL detection utilized the USLP 1.0 panel comprised of 1,536 SNP (Hyten et al., 2010) and a subset of SSR markers spanning ∼2,361 cM with an average distance of 1.0 cM across 20 soybean chromosomes. A total of 700 SNP and 204 SSR polymorphic markers were used to construct the linkage map (Vuong et al., 2011). The permutation test determined a significant LOD threshold of 2.3 for genome‐wide analysis. Two QTL were detected by MQM method (Table 2; Figure 1b). The first QTL explained 11.3% of total phenotypic variance and was mapped to Chr. 11 with the LOD score of 4.7 (Figure 1b). The additive effect of this QTL was 10.9. The 1.4‐Mb interval harboring this QTL was positioned between Sat_123 and BARC‐018869‐03031 markers that corresponded to Gm11:32194583‐33581636 (Wm82.a2.v1) physical interval. The second QTL was mapped to Chr. 18 between markers BARC‐055551‐13421 and BARC‐048275‐10534 covering the ∼397‐kb region that corresponded to a physical distance of Gm18:01308798‐01705500 (Figure 1b). The LOD peak reached 3.9, the QTL explained 7.3% of the total phenotypic variance, and its additive effect was 8.3.

**TABLE 2:** Summary of quantitative trait loci (QTL) mapping, marker intervals, significant logarithm of the odds (LOD) scores, *R* ^2^ values, and additive effects of QTL resistance to *Rotylenchulus reniformis* (RN) mapped on chromosomes. 3, 11, and 18 in ‘Magellan’ × PI 438489B recombinant inbred line population using two linkage maps

Genotyping method^a^	Chromosome (linkage group)	Confidence interval	Physical position^b^	LOD	*R* ^2^	Add^c^
			bp		%	
USLP 1.0	11 (B1)	Sat_123–BARC‐018869‐03031	32,194,583–33,581,636	4.7	11.3	10.9
	18 (G)	BARC‐055551‐13421–BARC‐048275‐10534	1,308,798–1,705,500	4.1	7.7	8.6
WGRS	3 (N)	bin151–bin152	39,781,662–40,299,533	3.3	5.3	‐6.6
	11 (B1)	bin122–bin124	32,892,556–32,978,390	5.5	9.3	9.1
	18 (G)	bin013–bin014	1,502,699–1,778,708	4.4	7.3	8.0

^a^ Genetic linkage map used for QTL mapping: USLP 1.0, universal soybean linkage panel; WGRS, whole‐genome resequencing.

^b^ Physical position in base pairs based on Williams 82 reference genome (Wm82.a2.v1).

^c^ Add, addiitive effect on the specific QTL.

The second approach of QTL detection used WGRS with 3,509 bins comprised of 9,296 SNPs spanning ∼2,964 cM with an average distance of 0.77 cM across 20 soybean chromosomes. The created bin linkage map was previously published by Xu et al. (2013). The permutation test determined a significant LOD threshold of 2.5 for genome‐wide analysis. Three QTL for RN resistance were detected using MQM (Table 2). The first QTL was mapped to Chr. 3 (Figure 1c). The interval of 518 kb harboring this QTL was situated between bin151 and bin152, which corresponded to a physical distance of Gm03:39781662‐40299533 (Wm82.a2.v1). The highest peak of this QTL was positioned at bin152 reaching LOD score of 3.3. This QTL explained 5.3% of the total phenotypic variance, and its additive effect was −6.7, suggesting Magellan to be a donor. The second QTL was mapped to Chr. 11 between bin122 and bin124 that corresponded to a physical distance of Gm11:32892556‐32978390 (85 kb) (Figure 1c). The QTL peak value of 5.5 was positioned at bin122; it explained 9.3% of the total phenotypic variation and displayed an additive effect of 9.1. The third QTL was mapped to Chr. 18 with LOD of 4.4 and it explained 7.3% of total phenotypic variance (Figure 1c). The confidence interval was located between bin013 and bin014 that translates to a physical distance of Gm18:1502699‐1778708 (276 kb). The additive effect of this QTL was 8.0, and bin014 (Gm18:1568360–1778708) was associated with the QTL peak.

### 3.2 Delineation of mapped QTL and pinpointing candidate genes

Detected QTL regions using the USLP 1.0 array and WGRS were compared with each other (Table 2) and previously reported QTL (Supplemental Tables S1 and S2). The confidence interval of QTL mapped on Chr. 11 using USLP 1.0 data overlapped with QTL detected using WGRS. Mapping resolution of the bin‐based map was much higher, and the genomic region was narrowed down to 86 kb that contained 10 candidate genes within the reference genome of Williams 82 (Wm82.a2.v1). This QTL was previously reported between Satt359 and Satt484 (Gm11:32411352‐33593871) in PI 437654 (Ha et al., 2007) and between Satt444 and BARC‐042299‐08241 (Gm11:29759358‐32433567) in PI 567516C (Jiao et al., 2015) (Supplemental Table S1). Interestingly, all RN resistance QTL detected on Chr. 11 overlapped with the previously reported SCN resistance QTL (Supplemental Table S1). The same results were observed for QTL mapped on Chr. 18, where the confidence interval obtained from USLP 1.0‐based mapping matched the region detected using WGRS mapping. The overlapped region was narrowed down to 276 kb and contained 28 candidate genes. The mapping power of both linkage maps was comparable at this locus. This QTL was previously reported to be linked to Sat_168 (Gm18:1706200) in PI 437654 (Ha et al., 2007) between BARC‐042717‐08388 and BARC‐048275‐10534 (Gm18:1232181‐1705500) in PI 567516C (Jiao et al., 2015) and to Satt275, Satt163, and Satt309 (Gm18:1240120‐1736779) in Hartwig (Lee et al., 2016) (Supplemental Table S2). All these QTL also overlapped with the *rhg1* locus that confers resistance to SCN (Supplemental Table S2). The QTL on Chr. 3 (Gm03:39781662‐40299533) was only detected using the WGRS approach because of the high‐resolution map composed of bins located in high density within the genome. Previously, the genomic region inherited from Magellan were not reported for RN resistance; however, SCN resistance QTL on Chr. 3 was mapped near A280Hae‐1 restriction fragment length polymorphism marker and between Sat_280 and Satt549 (Gm03:32670432‐37342918) in PI 404198A (Concibido, Lange, Denny, Orf, & Young, 1997; Guo, Sleper, Nguyen, Arelli, & Shannon, 2006).

Physical delineation of detected QTL produced physical intervals sufficiently small to warrant identification of candidate genes for RN resistance. The intervals of these QTL span potential genes that have been implicated in plant defense responses including receptor‐like protein kinases, wound‐induced proteins, and transcription factors (www.soybase.org) (Table 3). The QTL on Chr. 18 revealed a significant candidate gene, *Glyma.18g022500*, producing alpha‐soluble N‐ethylmaleimide‐sensitive factor attachment protein (SNAP) located at Gm18:1641008‐1645711 and will be further referred to as *GmSNAP18*. The locus was previously assigned as *rhg1* and has been reported to trigger resistance response to SCN (Bayless et al., 2016; Cook et al., 2012). Within QTL region on Chr. 11, the paralog of *GmSNAP18*, harboring resistance to SCN, has been located. The paralog *Glyma.11g234500* was located at Gm11:32967943‐32972705 and will be further referred to as *GmSNAP11*. Most interesting, SCN resistance locus *qSCN11* also overlapped with previous mapping efforts (Supplemental Table S1). The *qSCN11* was previously mapped between Satt583 and A006 (Gm11:9021577‐36660484) in PI 089772 (Yue, Sleper, & Arelli, 2001b) between Satt359 and Satt453 (Gm11:32411352‐34173264) in PI 090763 (Guo, Sleper, Arelli, Shannon, & Nguyen, 2005) linked to Satt453 (Gm11:34173240) in PI 404198A (Guo et al., 2006) and between Satt665 and EacaMctt067 (Gm11:31754150‐34273810) in PI 437654 (Wu et al., 2009). No paralogs of *GmSNAP18* (*rhg1*) and *GmSHMT08* (*Rhg4*) were found on Chr. 3 (Lakhssassi et al., 2017b), and it was not further analyzed in light of Magellan being a donor of this QTL.

**TABLE 3:** Summary of selected candidate genes identified in confidence intervals of quantitative trait loci detected using whole‐genome resequencing on chromosomes 3, 11, and 18 conferring resistance to reniform nematode (RN‐*Rotylenchulus reniformis*) in PI 438489B soybean accession

No.	Chromosome (linkage group)	Gene model^a^	Start	Stop	Putative function
			bp		
1	3 (N)	*Glyma.03g188000*	39,931,689	39,933,856	PLATZ transcription factor
2	3 (N)	*Glyma.03g188700*	39,976,660	39,978,543	Leucine‐rich repeat receptor‐like protein kinase
3	3 (N)	*Glyma.03g189400*	40,028,208	40,030,427	Transcription factor HEX with HOX and HALZ domains
4	3 (N)	*Glyma.03g189600*	40,045,932	40,048,046	Transcription factor GT‐2 with DNA‐binding/SANT domain
5	3 (N)	*Glyma.03g189700*	40,049,277	40,056,481	Pyruvate kinase
6	3 (N)	*Glyma.03g189800*	40,060,513	40,063,022	Leucine‐rich repeat receptor‐like protein kinase
7	3 (N)	*Glyma.03g191000*	40,178,777	40,191,743	Serine‐threonine protein kinase
8	3 (N)	*Glyma.03g191700*	40,237,382	40,240,885	Transcription factor GT‐2 with DNA‐binding/SANT domain
9	3 (N)	*Glyma.03g192100*	40,291,397	40,293,514	NTKL‐binging protein 1–transcriptional activator
10	11 (B1)	*Glyma.11g234300*	32,957,014	32,957,532	Wound‐induced protein WI12
11	11 (B1)	*Glyma.11g234500*	32,967,943	32,972,705	Soluble NSF attachment protein (SNAP)
12	18 (G)	*Glyma.18g020800*	1,510,384	1,515,694	Membrane associated ring finger protein
13	18 (G)	*Glyma.18g021600*	1,584,996	1,589,570	Serine–threonine protein kinase
14	18 (G)	*Glyma.18g022500*	1,641,008	1,645,711	Soluble NSF attachment protein (SNAP)
15	18 (G)	*Glyma.18g022700*	1,651,215	1,653,524	Wound‐induced protein WI12
16	18 (G)	*Glyma.18g023500*	1,712,011	1,715,223	Leucine‐rich repeat receptor‐like protein kinase

^a^ Gene models and their physical positions were assigned based on the Williams 82 reference genome (Wm82.a2.v1).

### 3.3 Sequence‐similarity clustering

**FIGURE 2:**
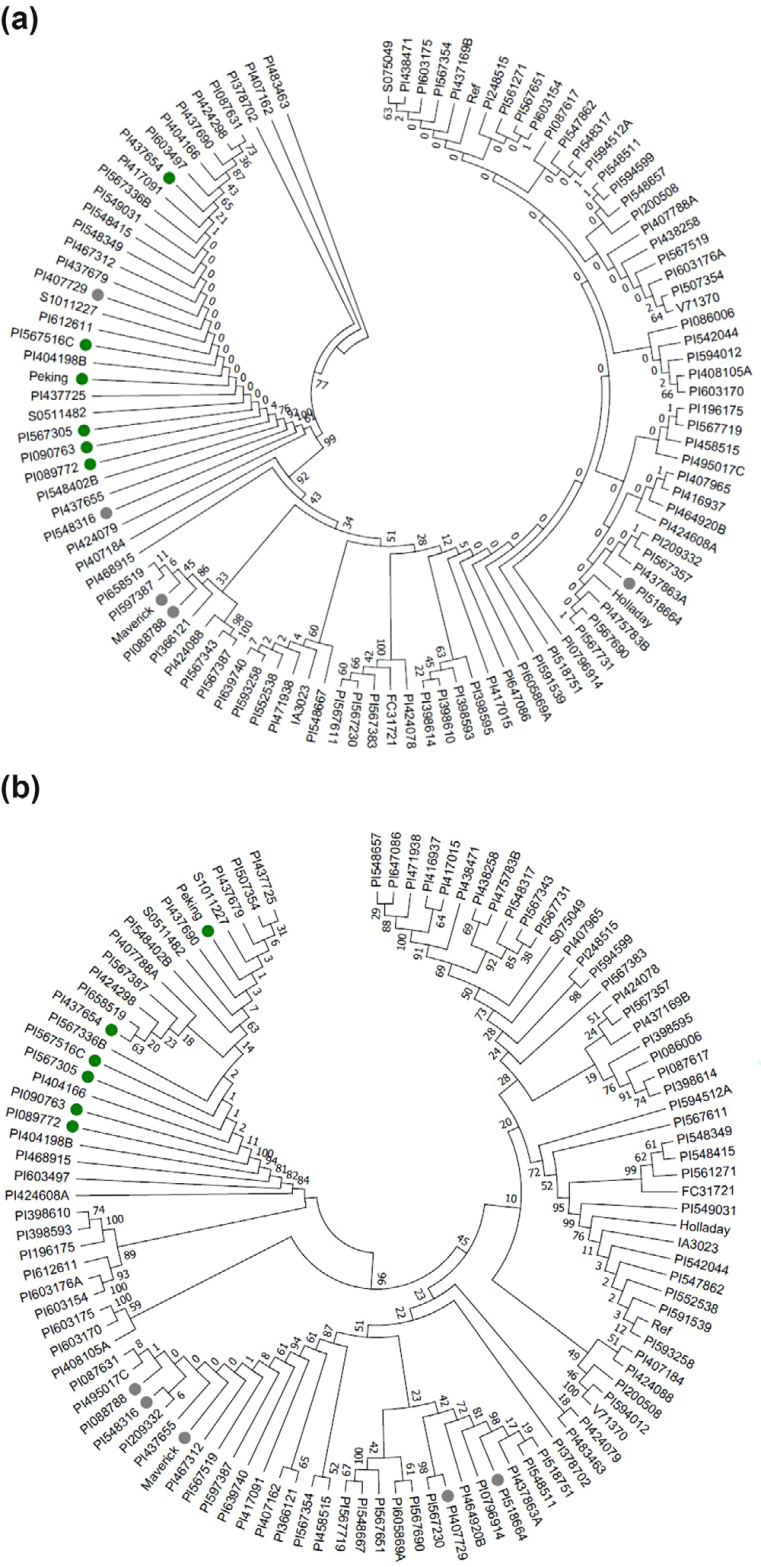
Sequence similarity clustering of 107 soybean accessions using the neighbor‐joining method: (a) clustering of 1,364 single nucleotide polymorphisms present in the interval Gm11: 32892556–32978390; (b) clustering of 3,594 SNPs present in the interval Gm18: 01502699–01778708. Reniform nematode resistant and susceptible checks were marked as green and grey dots, respectively

The confidence intervals of mapped QTL on Chrs. 11 and 18 using WGRS (Table 2) were used to retrieve WGRS‐based SNP call matrix comprised of 1,364 and 3,594 SNPs for QTL on Chrs. 11 and 18, respectively. The WGRS of 107 soybean accessions represented >96% of the sequence diversity (Valliyodan et al., 2016). The clustering was done on a single copy from each line knowing that *rhg1* can be present in up to 10 copies organized in tandem repeats. In good agreement with our previous report (Patil et al., 2019), no sequence variations were found within copies of high‐copy check accessions. To delineate the diversity of identified QTL on the Chr. 11 86‐kb region (Gm11:32892556‐32978390) was included in clustering. All resistant accessions clustered together; however, several susceptible lines were clustered separately in the following clade (Figure 2a). Similarly, for Chr. 18 QTL 276‐kb genomic region (Gm18:1502699‐1778708; 276 kb) was included in clustering. All accessions carrying Peking‐type *GmSNAP18* clustered together and displayed resistance to RN. It was noticeable that PI 88788‐type *GmSNAP18* clustered separately and it contained both RN resistant and susceptible accessions (Figure 2b).

### 3.4 Haplotype analysis and copy number variation of *GmSNAP11* and *GmSNAP18*

**FIGURE 3:**
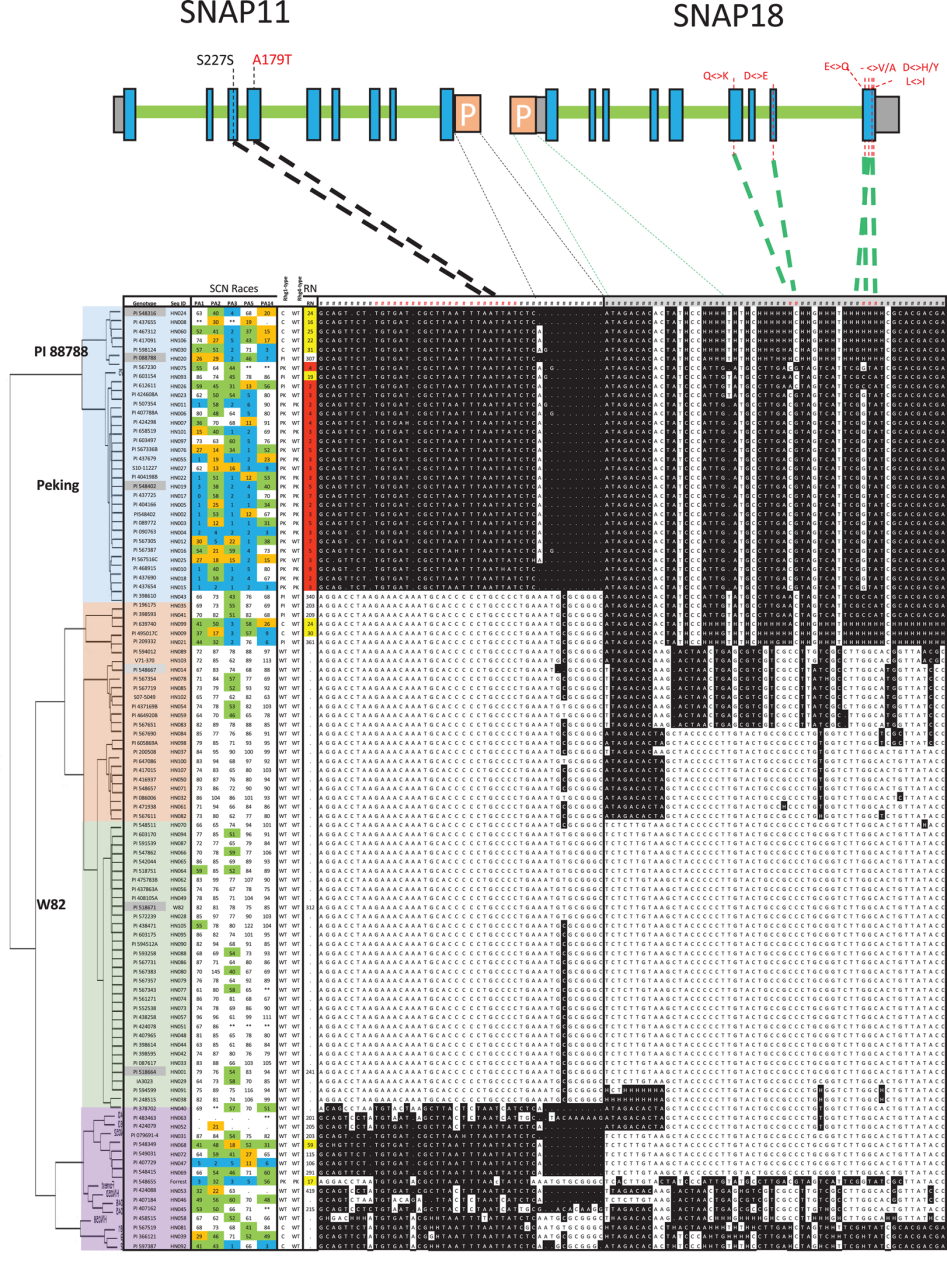
Haplotype analysis of *GmSNAP18* (*Glyma.18g022500*) and *GmSNAP11* (*Glyma.11g234500*) in the 107 diverse soybean lines. The haplotype clusters represent the association between soybean cyst nematode (SCN)–reniform nematode (RN) phenotypes with allelic variation at *GmSNAP18* and *GmSNAP11* coding or noncoding and promoter regions. In the schematic gene model (top of the haplotype cluster), the blue box represents exons, green bar represents introns, orange box represents promoter region and grey box represents 3′ or 5′ untranslated region. The single nucleotide polymorphisms (SNPs) in black background are different to the reference genome of Williams 82. The heterozygous SNPs are denoted as ‘H’ letter and deletions denoted by ‘.’. The nonsynonymous SNP leading to amino acid change are shown on the top of the gene model. HN numbers represent sequencing identification. Resistance to SCN reflected as the female index (FI) are shown for each genotype × SCN population (PA1, PA2, PA3, PA5, and PA14). Blue, orange, and green numbers represent resistance (FI < 10), moderate resistance (FI > 10 < 30), and moderate susceptibility (FI > 30 < 60) to SCN, respectively. Resistance to soybean RN was reflected as the reproductive index (RI) where the red and yellow numbers represent resistance and moderate resistance to RN, respectively. Haplotypes of *rhg1* and *Rhg4* were designated as C, Cloud‐type *rhg1‐b1*; PI, PI 88788‐type *rhg1‐b*; PK, Peking‐type *rhg1‐a* and *Rhg4‐a*; WT, susceptible wild‐type *rhg1‐c* and *Rhg4‐b* (Patil et al., 2019)

The genetic diversity at *GmSNAP11* and *GmSNAP18* genes provided information of the haplotype variation and their association with RN resistance. In a recent study (Patil et al., 2019), haplotype analysis of *GmSNAP18* was conducted, and nine nonsynonymous changes (amino acid sequence alteration) were reported leading to identification of three main haplotypes. In the present study, WGRS‐derived haplotype analysis of *GmSNAP11* and *GmSNAP18* was performed and the variants calls from both the genes were analyzed together to visualize their additive effect and infer the correlation with phenotype (Figure 3). Haplotype analysis revealed several synonymous and one nonsynonymous SNP at the *GmSNAP11* and displayed a strong association with RN resistance. The resistant allele contained a critical spontaneously occurring natural point mutation resulting in A179T amino acid change in exon 6 at the *GmSNAP11* protein when compared with susceptible or reference genome alleles. Interestingly, nine deletions were identified in the upstream promoter region, which was in strong association with RN resistance (Figure 3). Haplotype analysis collated with RN phenotyping of 52 soybean lines, suggesting that *GmSNAP11* and *GmSNAP18* could not work independently to obtain RN resistance; therefore, if only one of these genes has a resistant haplotype, the soybean line is susceptible to RN. Moreover, Peking‐type *GmSNAP18* (*rhg1‐a*) seems to have higher compatibility with *GmSNAP11* than PI 88788‐type (*rhg1‐b*) and Cloud‐type (*rhg1‐b1*) *GmSNAP18*, displaying a high level of resistance (RI < 10) to RN. Only two accessions PI 639740 and PI 495017C displayed moderate resistance to RN while missing the resistant allele of *GmSNAP11*. Peking‐type *GmSNAP18* (*rhg1‐a*) combined with *GmSNAP11* significantly scored high resistance, while PI 88788‐type *GmSNAP18* (*rhg1‐b*) combined with *GmSNAP11* displayed moderate resistance in most cases.

It was recently reported that two genes, *rhg1* (*GmSNAP18*) and *Rhg4* (*GmSHMT08*), are present in multiple copies associated with resistance to SCN (Cook et al., 2012; Patil et al., 2019). To test potential CNV of *GmSNAP11* and *GmSNAP18* and association between CNV and RN resistance, CGH was performed (Table 4). Among analyzed RN and SCN checks, *GmSNAP18* displayed CNV as expected, whereas *GmSNAP11* did not appear to show evidence of CNV. Further, to support this observation, NAM population data of CGH and WGRS was downloaded from the University of Minnesota database (http://stuparlabcnv.cfans.umn.edu:8080/). Among 41 NAM parents, seven accessions displayed CNV other than a single copy of *GmSNAP18*; whereas no CNV was observed for *GmSNAP11* (Supplemental Table S3).

**TABLE 4:** Comparison of *GmSNAP11* (*Glyma.11g234500*) and *GmSNAP18* (*Glyma.18g022500*) copy number variation (CNV) from representative soybean cyst nematode (SCN) and reniform nematode (RN) resistant lines using comparative genome hybridization (CGH)

			CNV–CGH		
Genotype	Name	Sequence ID	*GmSNAP11*	*GmSNAP18*	RN^a^
PI 548402	‘Peking’	HN019	1.00	2.72	R
PI 088788	–	HN020	1.33	7.60	S
PI 548316	‘Cloud’	HN024	–	–	MR
PI 438489B	–	–	1.13	2.68	R
PI 437654	–	HN015	1.02	2.71	R
PI 567516C	–	HN025	–	–	R
PI 090763	–	HN004	–	–	R
PI 089772	–	HN003	–	–	R
PI 548655	‘Forrest’	–	0.97	2.66	MR
PI 595362	‘Magellan’	–	–	0.97	S

^a^RN, reaction of soybean genotype to *Rotylenchulus reniformis* infection: R, resistant; MR, moderately resistant; S, susceptible.

### 3.5 Structural differences and dependency between *GmSNAP11* and *GmSNAP18*

Lakhssassi et al. (2017b) reported that both *GmSNAP* genes on Chrs. 11 and 18 belong to a very large inverted duplicated segment containing 84 additional conserved duplicated genes. To understand the structural differences between *GmSNAP11* and other *GmSNAP* paralogs, their promoter, intron, and exon architecture was compared (Supplemental Figure S1A). Among all analyzed genes, *GmSNAP14* and *GmSNAP02* were clustered in separate subclade near *GmSNAP18* and *GmSNAP11*; however, *GmSNAP09* did not cluster with the other *GmSNAP*s. In agreement with the findings reported by Lakhssassi et al. (2017b), structural variation between *GmSNAP11* and *GmSNAP18* was not found; similarly, no structural variation within sequenced susceptible or resistant lines was identified for individual *GmSNAP11* or *GmSNAP18* genes except SNP variation in the promoter and coding region (Figure 3). The alignment of amino acid sequences of all SNAPs displayed highest similarities between GmSNAP11 and GmSNAP18 proteins (Supplemental Figure S1B). This observation suggested that these proteins have highly conserved amino acid residues (92% identity); however, both genes might diverge to acquire a novel specialized function after recent whole‐genome duplication (13 million yr ago) event and occurrence of natural mutation (Lakhssassi et al., 2017b).

To test the relationship dependency between *GmSNAP11* and *GmSNAP18*, 16 RILs derived from an Essex × Forrest cross, containing various combinations of presence or absence of these genes, were phenotyped with RN population (Table 5). Among these lines, three RILs carried only *GmSNAP11*, five RILs carried only *GmSNAP18*, and five RILs carried both *GmSNAP11* and *GmSNAP18*. The results demonstrated that RN resistance is maintained when both resistant haplotypes are present, and any absence of one of them will cause loss of RN resistance. As expected, the presence of segregating *GmSHMT08* (*Rgh4*) did not have any effect on RN resistance.

**TABLE 5:** Summary of recombinant inbred lines (RILs) selected from the ‘Essex’ × ‘Forrest’ population, which carry different genes combinations of GmSNAP11, GmSNAP18, and GmSHMT08, and their responses to reniform nematode (RN‐*Rotylenchulus reniformis*) infection

		Genotype^a^			Phenotype^b^	
No.	RIL	*SNAP11*	*SNAP18*	*SHMT08*	RI	Score
1	ExF‐05	–	+	+	207	S
2	ExF‐07	+	+	+	8	R
3	ExF‐08	+	–	–	207	S
4	ExF‐12	+	+	–	7	R
5	ExF‐17	+	+	+	7	R
6	ExF‐20	–	+	–	131	S
7	ExF‐21	–	+	+	147	S
8	ExF‐23	–	+	+	159	S
9	ExF‐24	–	+	–	102	S
14	ExF‐26	+	–	–	197	S
13	ExF‐27	–	–	–	203	S
10	ExF‐29	+	+	+	8	R
15	ExF‐30	+	+	–	7	R
11	ExF 51	+	–	+	133	S
12	ExF 65	–	–	+	278	S
16	ExF‐68	–	–	+	211	S
17	‘Forrest’	+	+	+	8	R
18	‘Essex’	–	–	–	190	S
19	‘Hartwig’	+	+	+	4	R
20	‘Ellis’	–	–	–	241	S
–	Fallow	–	–	–	0	–

^a^ Haplotype classification of *GmSNAP11* and GmSNAP18: +, resistant haplotype, −, susceptible haplotype. The *GmSHMT08* (*Rhg4*) segregated in the RILs background; however, it does not seem to have an effect on RN resistance.

^b^ Reaction of soybean genotype to reniform nematode (RN‐*Rotylenchulus reniformis*) infection: Rreproductive index (RI) was calculated as the number of vermiform nematodes at test termination (Pf)/initial infestation density (Pi); R, resistant; S, susceptible.

### 3.6 Development of diagnostic markers

**FIGURE 4:**
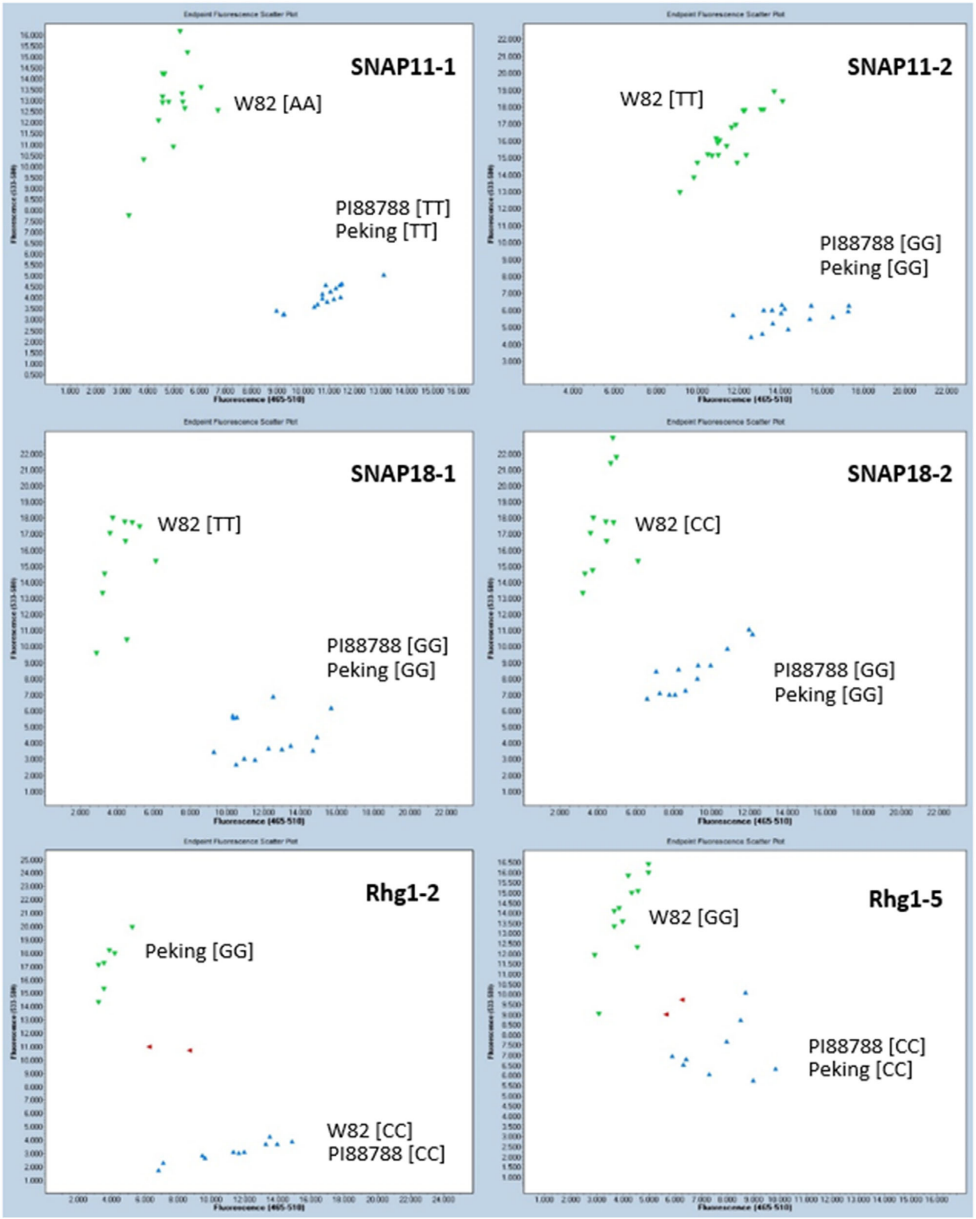
Endpoint fluorescence scattering plot of the Kompetitive allele specific polymerase chain reaction assays: *GmSNAP11‐1*, *GmSNAP11‐2*, *GmSNAP18‐1*, *GmSNAP18‐2*, *Rhg1‐2*, and *Rhg1‐5*, tested in a set of soybean nematode checks. Allele specific HEX primer (WT) was reported in green, allele‐specific FAM primer (mutant) was reported in blue, and heterozygous lines were marked in red. The *x* axis displays florescence of FAM at 523–568 nm, and the *y* axis displays fluorescence of HEX at 483–533 nm

The SNP matrix of *GmSNAP11* and *GmSNAP18* haplotype analysis was used to identify the SNPs associated with RN resistance that could be used to develop diagnostic assays applied to marker‐assisted selection (Table 5; Supplemental Table S4; Figure 4). Although the phenotype correlated on two closely related homologous loci, the markers were designed and validated on a set of RILs to be specific to a specific locus. Further validation of the assays was carried out in a set of nematode resistant or susceptible checks (Table 6). Two markers, SNAP11‐1 and SNAP11‐2, located in the *GmSNAP11* gene were developed to differentiate RN susceptible allele of Williams 82‐type (W82‐type) from resistant allele of PI 88788, Cloud, and Peking‐types. Marker SNAP11‐1 displayed slightly better separation of the samples, where the alleles were marked in blue (TT and GG for SNAP11‐1 and SNAP11‐2, respectively) to signify RN resistance (Figure 4).

**TABLE 6:** Performance of developed single nucleotide polymorphism (SNP) markers for soybean resistance to reniform nematode (RN‐*Rotylenchulus reniformis*) infection

Haplotype^a^	Genotype	Name	RN^b^	SNAP11‐1^c^	SNAP11‐2^c^	SNAP18‐1^c^	SNAP18‐2^c^	Rhg1‐2^d^	Rhg1‐5^d^
Williams82‐type	PI 595362	‘Magellan’	S	AA	TT	TT	CC	CC	GG
	PI 518671	‘Williams 82’	S	AA	TT	TT	CC	CC	GG
	PI 548667	‘Essex’	S	AA	TT	TT	CC	CC	GG
	PI 518664	‘Hutcheson’	S	AA	TT	TT	CC	CC	GG
	PI 548659	‘Braxton’	S	AA	TT	TT	CC	CC	GG
	PI 680630	‘Ellis’	S	AA	TT	TT	CC	GG	GG
PI88788‐type	PI 088788	–	S	TT	GG	GG	GG	CC	CC
	PI 209332	–	S	AA	TT	GG	GG	CC	CC
	PI 612611	–	MR	TT	GG	GG	GG	CC	CC
Cloud‐type	PI 548316	‘Cloud’	MR	TT	GG	GG	GG	CC	CC
	PI 598124	‘Maverick’	MR	TT	GG	GG	GG	CC	CC
Peking‐type	PI 548402	‘Peking’	R	TT	GG	GG	GG	GG	CC
	PI 438489B	–	R	TT	GG	GG	GG	GG	CC
	PI 089772	–	R	TT	GG	GG	GG	GG	GG
	PI 548655	‘Forrest’	MR	TT	GG	GG	GG	GG	CC
	PI 090763	–	R	TT	GG	GG	GG	GG	CC
	PI 567516C	–	R	TT	GG	GG	GG	GG	CC
	PI 567305	–	R	TT	GG	GG	GG	GG	CC
	PI 437654	–	R	TT	GG	GG	GG	GG	CC
	PI 543795	‘Hartwig’	R	TT	GG	GG	GG	GG	CC
	PI 614732	‘Anand’	R	TT	GG	GG	GG	GG	CC

^a^ *GmSNAP18* haplotype classification of soybean cyst nematode (SCN) response (Patil et al. 2019).

^b^ Reaction of soybean genotype to *Rotylenchulus reniformis* infection: R, resistant; MR, moderately resistant; S, susceptible.

^c^ Markers developed in this study for RN testing: SNAP11‐1 (Gm11:32968127) and SNAP11‐2 (Gm11:32970557) for testing *GmSNAP11*, and SNAP18‐1 (Gm18:1645012) and SNAP18‐2 (Gm18:1643107) for testing *GmSNAP18*.

^d^ Markers Rhg1‐2 (Gm18:1643660) and Rhg1‐5 (Gm18:1645402) developed for SCN testing of *GmSNAP18* (Kadam et al., 2016).

The two markers, SNAP18‐1 and SNAP18‐2 identified in this study, showed similar separation of soybean checks with the previously known marker Rhg1‐2 that differentiate W82 type from PI 88788 and Cloud and Peking types (Kadam et al., 2016). These markers can work interchangeably; however, there was a better separation of samples while using SNAP18‐1 (Figure 4). The alleles marked in blue in Figure 4 (GG, GG, and CC for SNAP18‐1, SNAP18‐2, and Rhg1‐5, respectively) signify RN resistance. The previously developed marker, Rhg1‐2 (Kadam et al., 2016), separated Peking‐type from W82 and PI88788‐type of *GmSNAP18*; therefore, it has a more significant function in SCN rather than RN screening. However, Rhg1‐2 marker is able to detect Peking‐type of *GmSNAP18* that seem to play a role in extreme resistance to RN (Table 6; Figures 3 and 4). It is important to note that screening for RN resistance will require using markers for both genes simultaneously because RN resistant alleles from both genes *GmSNAP11* and *GmSNAP18* need to be present to confer resistance to RN (Figure 3).

### 3.7 Homology modeling of GmSNAP18 and GmSNAP11 predict a putative interaction site

**FIGURE 5:**
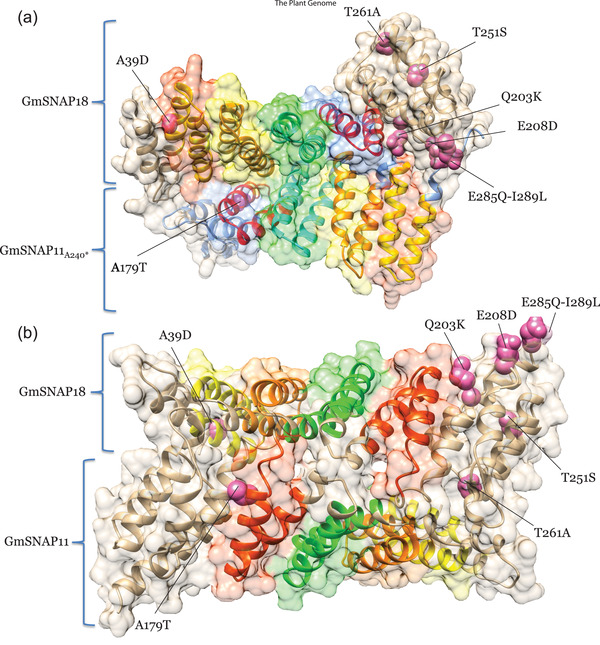
Homology modelling of the GmSNAP18, GmSNAP11, and GmSNAP11_A240*_ from Forrest (Peking‐type resistance). (a) The predicted GmSNAP18×GmSNAP11_A240*_ interaction. (b) The predicted GmSNAP18×GmSNAP11 interaction. Both interactions were predicted to form a dimer. The highlighted pink residues correspond to the different reported haplotypes between soybean cyst nematode and reniform nematode resistant and susceptible lines (five and one highlighted residues at the GmSNAP18 and GmSNAP11, respectively). Protein homology modeling and docking algorithms employed predicted the presence of an interaction between GmSNAP18 × GmSNAP11 and GmSNAP18 × GmSNAP11_A240*_. Locations of the tetratricopeptide repeat (TPR) domain and haplotypes of GmSNAP18 are shown. TPR1, TPR2, TPR3, and TPR4 motifs are highlighted in yellow, orange, green, and red respectively. The interaction between GmSNAP18 and at GmSNAP11 or GmSNAP11_A240*_ is predicted to involve all four TPR domains in addition to the C‐terminal of the GmSNAP18 protein, which carries most of the reported haplotypes. The two other haplotypes were mapped at the first TPR motif of the GmSNAP18 (A39D), and at the fourth TPR motif of the GmSNAP11 or GmSNAP11_A240*_ (A179T). The presence of these haplotypes is predicted to play an essential role on pathogen resistance and may impact the interaction between the GmSNAP18 and GmSNAP11 or GmSNAP11_A240*_ proteins

The SNAP genes carry several key domains where the TPR domains facilitate specific interactions with partner proteins (Blatch & Lassle, 1999). To predict a putative interaction site between both proteins, homology modeling was carried out using GmSNAP18 and the two types, GmSNAP11 and GmSNAP11_A240*_, of sequences from Forrest. Next, protein–protein docking algorithms were implemented. Interestingly, the location of the putative interaction site was predicted to involve the four TPR motifs at both the GmSNAP18 and GmSNAP11_A240*_ (Figure 5a) or GmSNAP11 proteins (Figure 5b) to form a dimer. Protein homology modeling predicted that the polymorphism A179T present between susceptible and resistant soybean lines was found within the interaction site between both proteins involving the TPR4 motif at the GmSNAP11 or GmSNAP11_A240*_ and the TPR2 motif at the GmSNAP18. Moreover, the last five amino acids at the GmSNAP18, in addition to the E208D and Q203K polymorphisms between susceptible and resistant soybean lines, was predicted to impact the interaction between the C‐terminal of the GmSNAP18 and the TPR1 motif at the GmSNAP11_A240*_. The T261A polymorphism may impact the interaction between the C‐terminal of the GmSNAP18 and the TPR1 motif at the GmSNAP11 (Figure 5b).

The other polymorphic site, A39D at the GmSNAP18, was located on the β‐strand that holds an arm protruding out of GmSNAP18 and is predicted to impact the interaction with C‐terminal at the GmSNAP11 or GmSNAP11_A240*_ proteins (Figure 5). In vitro protein interaction studies are necessary to determine the effect of the sequence differences on the affinity of the two proteins.

### 3.8 GmSNAP18 and GmSNAP11 proteins interact at the cellular level

**FIGURE 6:**
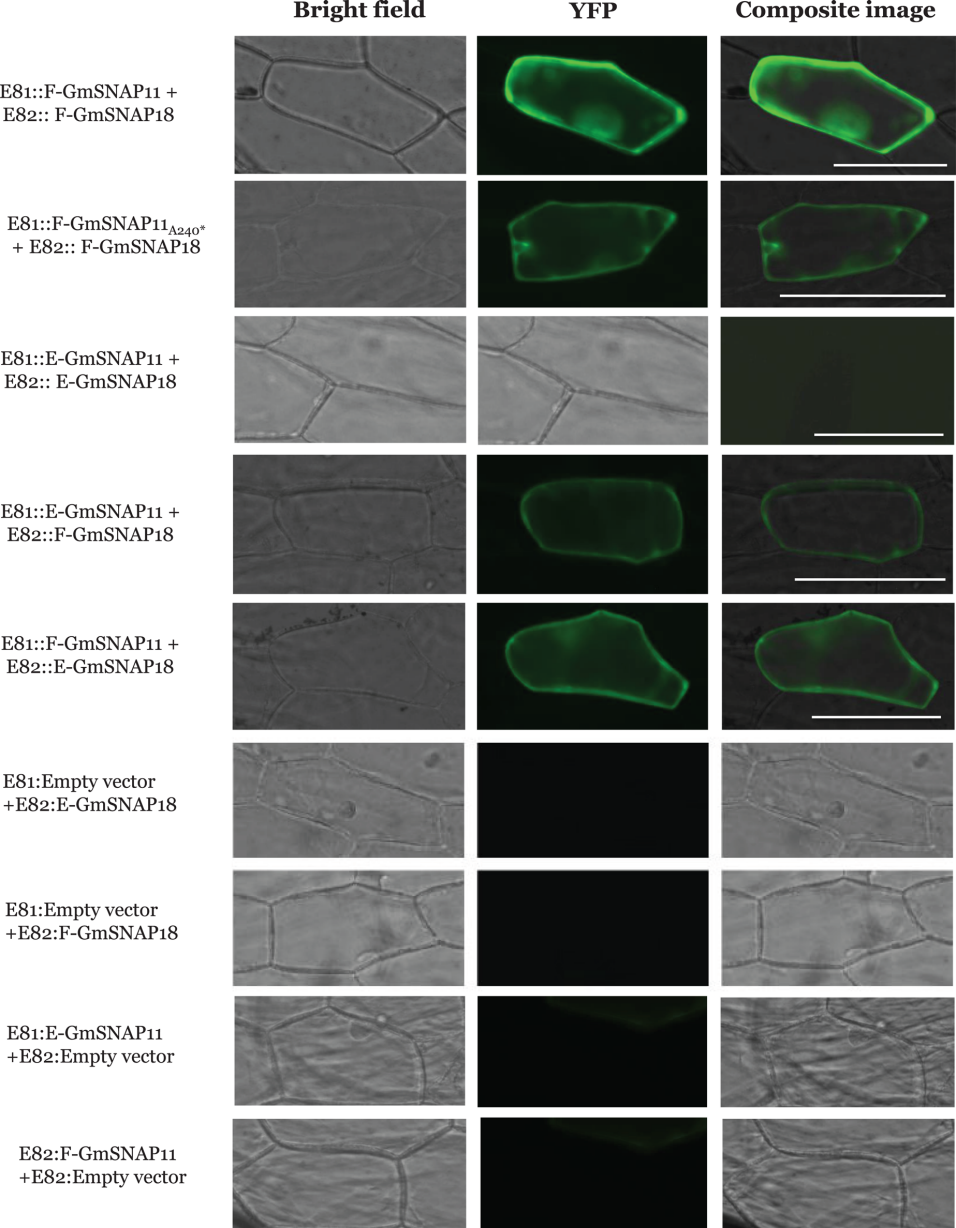
Bimolecular fluorescence complementation analysis between GmSNAP18 and GmSNAP11 or GmSNAP11_A240*_. The coding sequences of Forrest and Essex *GmSNAP11* and GmSNAP11_A240*_ were cloned into *pSAT4‐nEYFP‐C1* (E81) to generate *nEYFP‐GmSNAP11* and *nEYFP‐*GmSNAP11_A240*_ fusions. Likewise, *GmSNAP18* coding sequences from Forrest and Essex wild‐types were cloned into *pSAT4‐cEYFP‐C1‐B* (E82) to generate *cEYFP‐GmSNAP18* fusions. Various combinations of cEYFP and nEYFP fusions including negative controls were coexpressed in onion epidermal cells by particle bombardment. Interactions were stronger when both the GmSNAP18^+^ and GmSNAP11^+^ were present as resistant alleles. While using the truncated GmSNAP11_A240*_, intensity and number of interacting cells decreased (one cell only). However, weak interactions were observed when at least one of the two partners is present as susceptible allele. Interestingly, when both proteins are present as susceptible allele (Essex), no interaction was observed. Bar = 200 μM

In Peking‐type resistance, it has been reported recently that GmSNAP18 forms a multi‐protein complex with two other partners—the GmSHMT08 and the GmPR08‐Bet VI proteins (Lakhssassi et al., 2020a). Bimolecular fluorescence complementation assay was carried out to gain more insight into the predicted interaction between the GmSNAP11 and GmSNAP18. A combination of different constructs expressing *pSAT4‐nEYFP‐C1::GmSNAP11*, pSAT4‐nEYFP‐C1:: GmSNAP11_A240*_, and *pSAT4‐cEYFP‐C1‐B::GmSNAP18* from both Essex and Forrest were used to check for any possible interaction. Surprisingly, the results obtained showed the presence of a strong interaction between the GmSNAP18 and GmSNAP11 proteins at the plasma membrane when both are present as resistant Forrest allele (more than 10 cells) (Figure 6). While using the GmSNAP11_A240*_, the number of interacting cells decreased significantly (one cell only). However, weak interactions were observed when at least one of the two partners were present as susceptible alleles (Figure 6). Interestingly, when both proteins are present as susceptible alleles, no interaction was observed. These findings support the hypothesis that GmSNAP11 is another partner of the GmSNAP18 protein, which is involved in the pathway for resistance to soybean cyst nematode.

## 4 DISCUSSION

Soybean line PI 438489B was identified as an excellent source of resistance to multiple SCN races and its QTL were mapped on Chrs. 1, 4, 5, 6, 8, 11, 14, 15, and 18 (Vuong et al., 2011; Yue, Arelli, & Sleper, 2001a). It was also reported that this PI contains three Peking‐type copies of the *GmSNAP18* gene (Klepadlo et al., 2018; Patil et al., 2019). Furthermore, PI 438489B harbors resistance to root‐knot nematode (RKN, *Meloidogyne incognita*) on Chrs. 8, 10, and 13 (Xu et al., 2013). In this study, PI 438489B was reported to be an excellent source of resistance to RN on Chrs. 11 and 18, thus carrying multi‐nematode resistance gene package valuable for soybean breeding. Using the USLP 1.0 panel combined with an extra set of SSR markers and Illumina next‐generation sequencing method makes this study more secure than most reporting the phenotype correlated on two closely related homoeologous loci, which are easy to conflate because of cross‐reactive markers.

The genetic association between SCN and RN resistance was reported for the first time in 1969, while no relationship was established between RN and RKN resistance in soybean (Rebois et al., 1968). Since that time, no research has reported evidence of association between SCN and RN resistance controlled by pleiotropic effects of the same resistance gene or linked different genes; however, some researchers indicated the presence of that possibility (Rebois et al., 1968; Ha et al., 2007; Klepadlo et al., 2018). This study indicates that *GmSNAP18* gene could control resistance to both SCN and RN at the *rhg1‐a* and *rhg1‐b* and *rhg1‐b1* allele. The paralog of this gene, *GmSNAP11* (Lakhssassi et al., 2017b), also plays a role in controlling resistance to both SCN and RN at *qSCN11* locus. Epistasis analysis will be needed to test genetic interactions between these two genes. This genetic association can be explained by formation of feeding sites in soybean roots that are similar to those of SCN (syncytium) and RN, and quite different from that of RKN (Rebois, Epps, & Hartwig, 1970). Recently, Lakhssassi et al. (2017b) reported that *GmSNAP11* plays a minor function in resistance to SCN as a result of its truncated protein.

It was previously reported that *GmSNAP18* gene at the *rhg1* locus and the *GmSHMT08* gene at the *Rhg4* locus are associated with SCN resistance based on copy number variations (Bayless et al., 2016; Cook et al., 2012; Patil et al., 2019). Although *GmSNAP18* exists in multiple copies, its paralog *GmSNAP11* displayed a single copy throughout all tested RN resistant lines and NAM parents. Nevertheless, a significant correlation in RN resistance was observed between the *rhg1* copy number of Peking‐type *GmSNAP18* (*rhg1‐a*) and PI 88788–Cloud‐type *GmSNAP18* (*rhg1‐b*). Both genes, *GmSNAP18* and *GmSNAP11*, seem to have an effect on resistance to both SCN and RN. The SCN locus *qSCN11* causes resistant reaction to SCN races 2 and 5 (Guo et al., 2005, 2006; Vuong et al., 2011; Yue at al., 2001a, 2001b), different from PI 88788‐type *rhg1* (SCN races 3 and 14) and Peking‐type *rhg1* + *Rhg4* (SCN races 1, 3, and 14) (Klepadlo et al., 2018, Patil et al., 2019). PI 438489B carries resistant haplotypes at *GmSNAP11* and Peking‐type *GmSNAP18* similar to PI 437654, PI 89772, and PI 90763, in which QTL for SCN resistance was detected on Chrs. 11 and 18. This phenomenon raises many questions about the differences and specificity in reactions to SCN and RN based on *GmSNAP11* structure and intergenic sequences and its relationship with *GmSNAP18*. Because QTL for resistance to SCN and RN were consistently mapped at *GmSNAP18* and *GmSNAP11* regions in all previously analyzed soybean lines, *GmSNAP18* might be a major gene, which works as ‘switch‐on’ signal for RN resistance, while *GmSNAP11* may cause a quantitative response.

The findings of this study enable us to comprehensively elucidate genetic and genomic aspects of SCN and RN resistance in PI 438489B; however, they also bring up a few more considerations. Previous researchers were misled to declare that the *rhg1* locus could cause resistance to RN because of the susceptible reaction of PI 88788 (Davis et al., 1996; Klepadlo et al., 2018; Robbins & Rakes, 1996; Stetina et al., 2014). However, the susceptibility of PI 88788 appears to be an exception because this accession carries both resistant haplotypes at *GmSNAP18* and *GmSNAP11*. There was no structural difference found that could explain this phenomenon; however, the genetic background, novel genes, or *trans*‐regulatory elements might have a role in this reaction, and it requires further molecular investigations. Moderately resistant RN lines PI 639740 and PI 495017C displayed the absence of the *GmSNAP11* resistant allele. This could be explained by the presence of different or novel genes that could contribute to RN resistance together with *GmSNAP18* including another *GmSNAP18* paralog. In addition, if *GmSNAP11* is the *qSCN11* locus that confers resistance to SCN, then this could be why it was detected using conventional QTL mapping in some occasions while it contains resistant *GmSNAP11* haplotype. Interestingly, in an earlier study (Vuong et al., 2011), the SCN resistance at *qSCN11* was not detected in the same Magellan × PI 438489B RIL population; however, Yue et al. (2001a) detected this QTL in PI 438489B × ‘Hamilton’ population. Moreover, mapping SCN and RN resistance QTL from accession PI 567516C using the same population displayed SCN resistance associated with Chrs. 10 and 18 (different location than *rhg1*) and RN resistance QTL mapped to Chrs. 11 and 18 that correspond to the location of *GmSNAP11* and *GmSNAP18*. This indicates that the resistant haplotypes might be present in a specific genotype but may not be active when attacked by a specific nematode. The other question is why the *qSCN11* locus is naturally present only in PIs that carry Peking‐type *GmSNAP18*? Finally, why does Peking‐type *GmSNAP18* in combination with *GmSNAP11* cause high resistance to RN, whereas PI 88788‐type *GmSNAP18* in combination with *GmSNAP11* causes only moderate resistance? There is a possibility of tightly linked genes responsible for resistance to SCN and RN in the soybean genome or diversification of some copies of *GmSNAP18*.

Soybean has a paleopolyploid genome with 75% of predicted genes present in multiple copies because of two duplication events (Schmutz et al., 2010). In soybean, the *SNAP* gene family is composed of five members: *Glyma.09g279400*, *Glyma.02g260400*, *Glyma.14g054900*, *Glyma.11g234500*, and *Glyma.18g022500* (Lakhssassi et al., 2017b). Among all *GmSNAP*s, the protein sequence alignment showed the highest similarity between *GmSNAP18* and *GmSNAP11*; in addition, both genes have highly conserved carboxylate clamp residues at four TPR domains (Lakhssassi et al., 2017b). It is possible that *GmSNAP11* and *GmSNAP18* derived from segmental duplication followed by tandem duplications of *GmSNAP18*. Most significantly, this study shows the presence of a physical interaction between the GmSNAP18 and GmSNAP11 proteins, suggesting that they belong to the same molecular pathway for resistance to nematodes. In a recent study by Lakhssassi et al. (2017b), natural nonsense mutations in *GmSNAP11* at position A240* resulted in the truncated protein GmSNAP11_A240*_, which may affect and reduce the function in Peking‐type Forrest without entirely suppressing its function. These data are coherent with the decreased interaction observed between both the GmSNAP18 and GmSNAP11_A240*_ (one cell only) when compared with the strongest interaction observed between the GmSNAP18 and GmSNAP11 proteins (more than 10 cells) (Figure 6). Unlike the *GmSHMT* gene family when only the GmSHMT08 has been shown to play a role in SCN resistance (Lakhssassi et al., 2019), *GmSNAP11* and *GmSNAP18* members show evidence of functional redundancy for resistance to both nematodes. The altered structure of both proteins could lead to their functional diversification gaining new molecular activities and novel biological functions, which support the neofunctionalization of the *GmSNAP* gene family members reported previously (Lakhssassi et al., 2017b). Moreover, it has been reported that the SCN genome is complex and carries sequence repeats, tandem duplications, and also experienced the horizontal gene transfer events involved in the evolution of parasitism genes (Masonbrink et al., 2018). Comparison of the SCN genome to the RN genome could answer questions related to the evolutionary mechanisms responsible for the emergence and diversification of effector genes; however, the RN genome has not been sequenced yet.

The power of natural selection depends on gene's pleiotropic effects on traits related to fitness (Williams, 1957). Directional pleiotropy is common in plant breeding because of selection applied to one trait that causes selection of another trait. Pleiotropy observed in the analyzed *GmSNAP* genes caused directional natural selection desirable for the breeder. Breeding for RN resistance has always been a lower priority relative to the efforts dedicated for SCN resistance in soybean; however, the continuous breeding for SCN resistance has caused unconscious selection for RN resistance over time. The QTL, molecular markers, and candidate genes identified in this study will accelerate the process of developing competitive SCN and RN resistant varieties. Continuous efforts of cotransformation and uncovering molecular mechanisms underlying RN resistance in soybean are highly suggestive.

## AUTHOR CONTRIBUTIONS

Mariola Usovsky: Conceptualization; Formal analysis; Investigation; Validation; Writing‐original draft; Writing‐review & editing. Naoufal Lakhssassi: Conceptualization; Formal analysis; Investigation; Methodology; Validation; Writing‐review & editing. Gunvant Patil: Formal analysis; Investigation; Software; Writing‐review & editing. Tri Vuong: Data curation; Formal analysis; Methodology; Resources; Validation; Writing‐review & editing. Sarbottam Piya: Formal analysis; Investigation; Methodology. Tarek Hewezi: Formal analysis; Investigation; Methodology; Supervision; Validation; Writing‐review & editing. Robert T. Robbins: Formal analysis; Investigation; Methodology; Supervision; Writing‐review & editing. Robert M. Stupar: Formal analysis; Investigation; Supervision; Writing‐review & editing. Khalid Meksem: Conceptualization; Data curation; Formal analysis; Funding acquisition; Investigation; Methodology; Project administration; Supervision; Validation; Writing‐review & editing. Henry T. Nguyen: Conceptualization; Data curation; Formal analysis; Funding acquisition; Investigation; Methodology; Project administration; Resources; Software; Supervision; Validation; Visualization; Writing‐review & editing.

## CONFLICT OF INTEREST

The authors declare that they have no conflict of interest.

## Supporting information




**Supplemental Figure S1**. Comparative analysis of five *GmSNAP* paralogs: A. Phylogenetic relationship and schematic representation of gene architecture; B. Amino acid alignment of SNAP proteins showing high amino acid residue conservation.

**Supplemental Figure S2**. Cloning the Forrest *GmSNAP11* in *pSAT4‐nEYFP‐C1* (E81) vector.

**Supplemental Figure S3**. Cloning the Forrest GmSNAP11_A240*_ in *pSAT4‐nEYFP‐C1* (E81) vector.

**Supplemental Figure S4**. Cloning the Essex *GmSNAP11* in *pSAT4‐nEYFP‐C1* (E81) vector.

**Supplemental Figure S5**. Cloning the Essex *GmSNAP18* in *pSAT4‐cEYFP‐C1‐B* (E82) vector.

**Supplemental Figure S6**. Cloning the Forrest *GmSNAP18* in *pSAT4‐cEYFP‐C1‐B* (E82) vector.

**Supplemental Table S1**. Comparison of physical position of *GmSNAP11* gene (orange) with RN resistance QTL on Chr.11 (green) and SCN resistance *qSCN11* (blue) reported in this and previous studies.

**Supplemental Table S2**. Comparison of physical position of *GmSNAP18* gene at the *rhg1* locus (orange) with RN resistance QTL on Chr.18 (green) reported in this and previous studies.

**Supplemental Table S3**. Estimation of CNV of *GmSNAP18* and *GmSNAP11* using comparative genome hybridization (CGH) and whole genome re‐sequence (WGRS) in NAM population. The data was accessed from the Stupar Lab, the University of Minnesota, MN (http://stuparlabcnv.cfans.umn.edu:8080/).

**Supplemental Table S4**. Primer sequences for diagnostic SNP assays detecting *Rotylenchulus reniformis* (RN) resistance.

Supplemental Table S5. Phenotypic data of mapping population (Magellan x PI 438489B); 247 recombinant inbred lines (RIL); Reniform nematode reproduction index (RI)

Supplemental Table S6. Linkage Map of mapping population (Magellan x PI 438489B); 247 recombinant inbred lines (RIL); markers: 3509 bins
