# Supplementary material for: Dissecting nematode resistance regions in soybean revealed pleiotropic effect of soybean cyst and reniform nematode resistance genes
Source: Plant Genome. 2021 Mar 16;14(2):e20083. doi: 10.1002/tpg2.20083 (PMC12807396; doi:10.1002/tpg2.20083)
Supplement: Supplementary file 1 [file TPG2-14-e20083-s001.pdf]

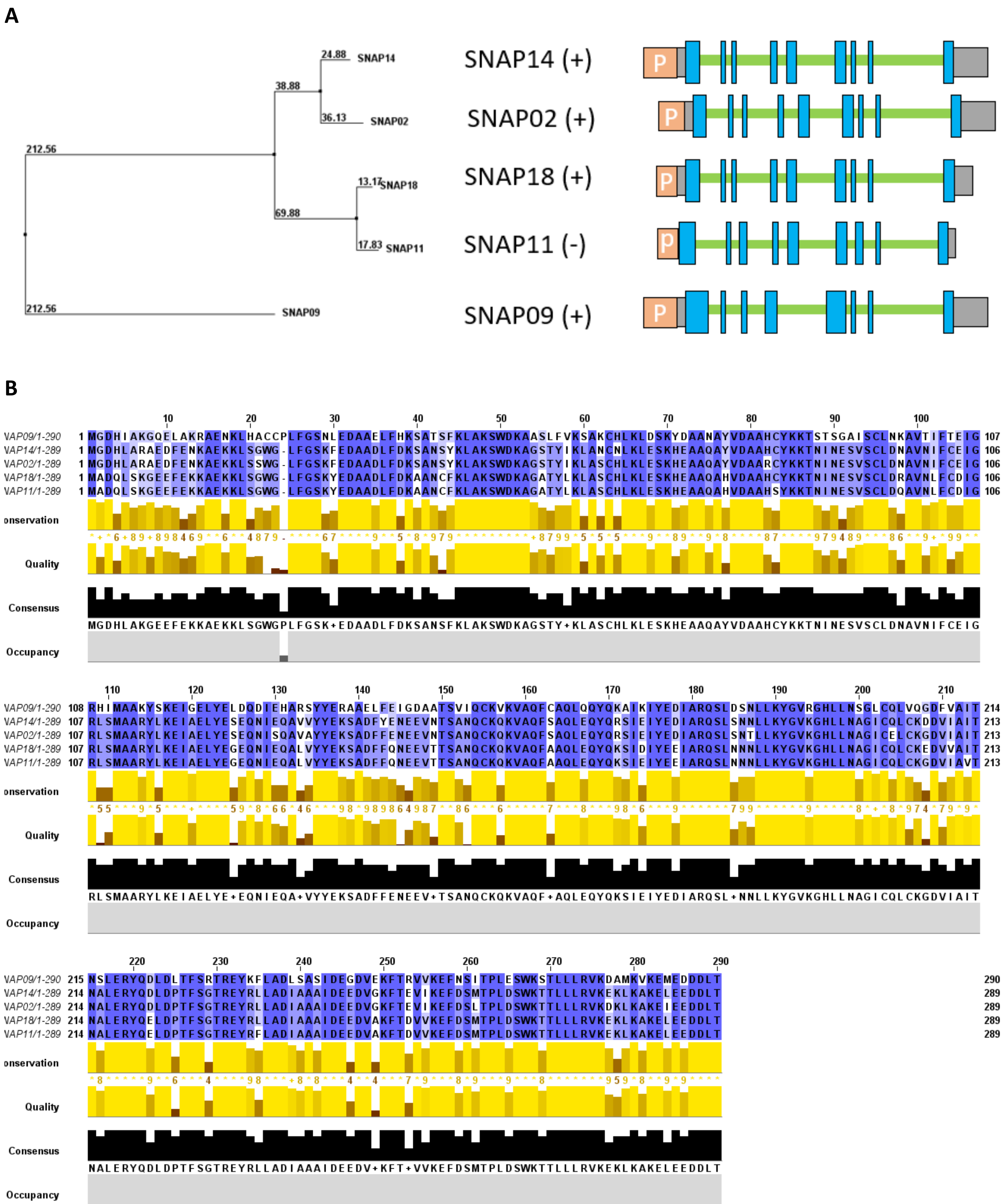

Figure S1. Comparative analysis of five *GmSNAP* paralogs: A. Phylogenetic relationship and schematic representation of gene architecture; B. Amino acid alignment of SNAP proteins showing high amino acid residue conservation.
