# Supplementary material for: Dissecting nematode resistance regions in soybean revealed pleiotropic effect of soybean cyst and reniform nematode resistance genes
Source: Plant Genome. 2021 Mar 16;14(2):e20083. doi: 10.1002/tpg2.20083 (PMC12807396; doi:10.1002/tpg2.20083)
Supplement: Supplementary file 2 [file TPG2-14-e20083-s002.pdf]

Gene Name F-SNAP11-full CDS
Cloning Vector *pSAT4-nEYFP-C1 (E81)*
Length (bp) 884
Cloning Sites HindIII(AAGCTT)-BamHI(GGATCC)

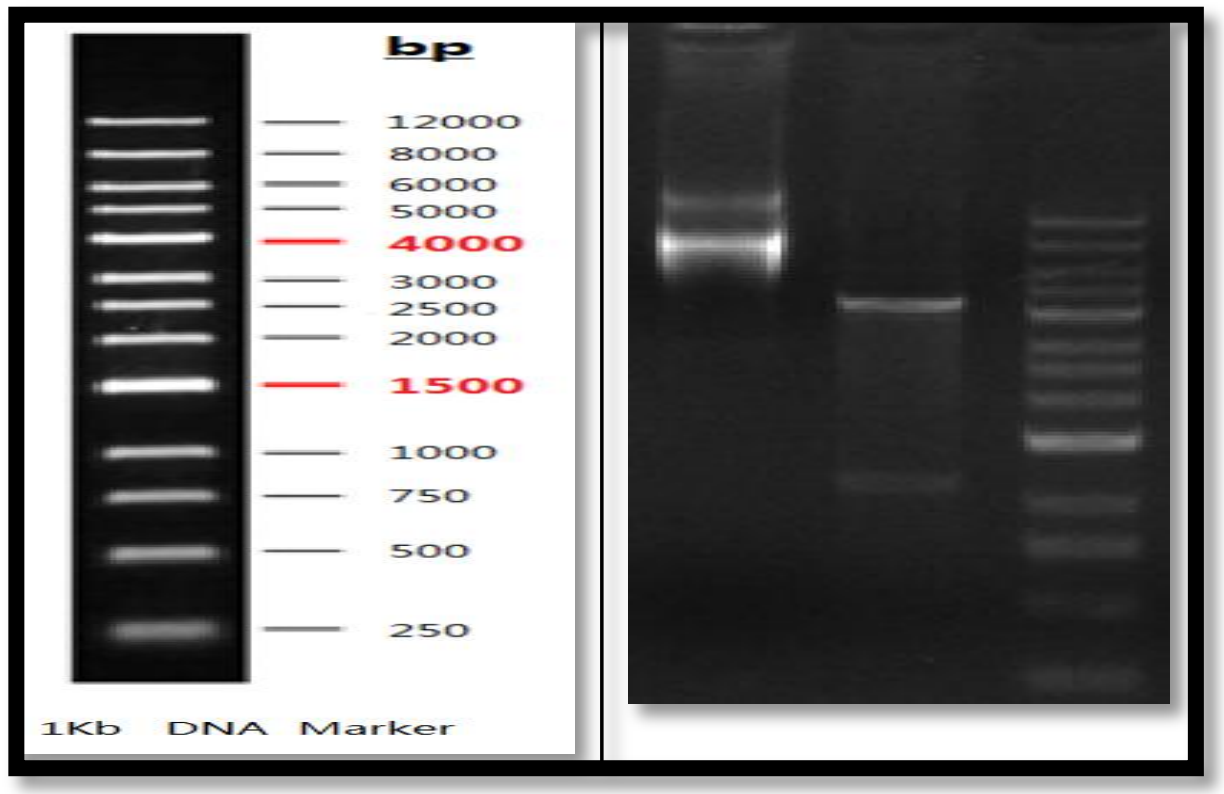

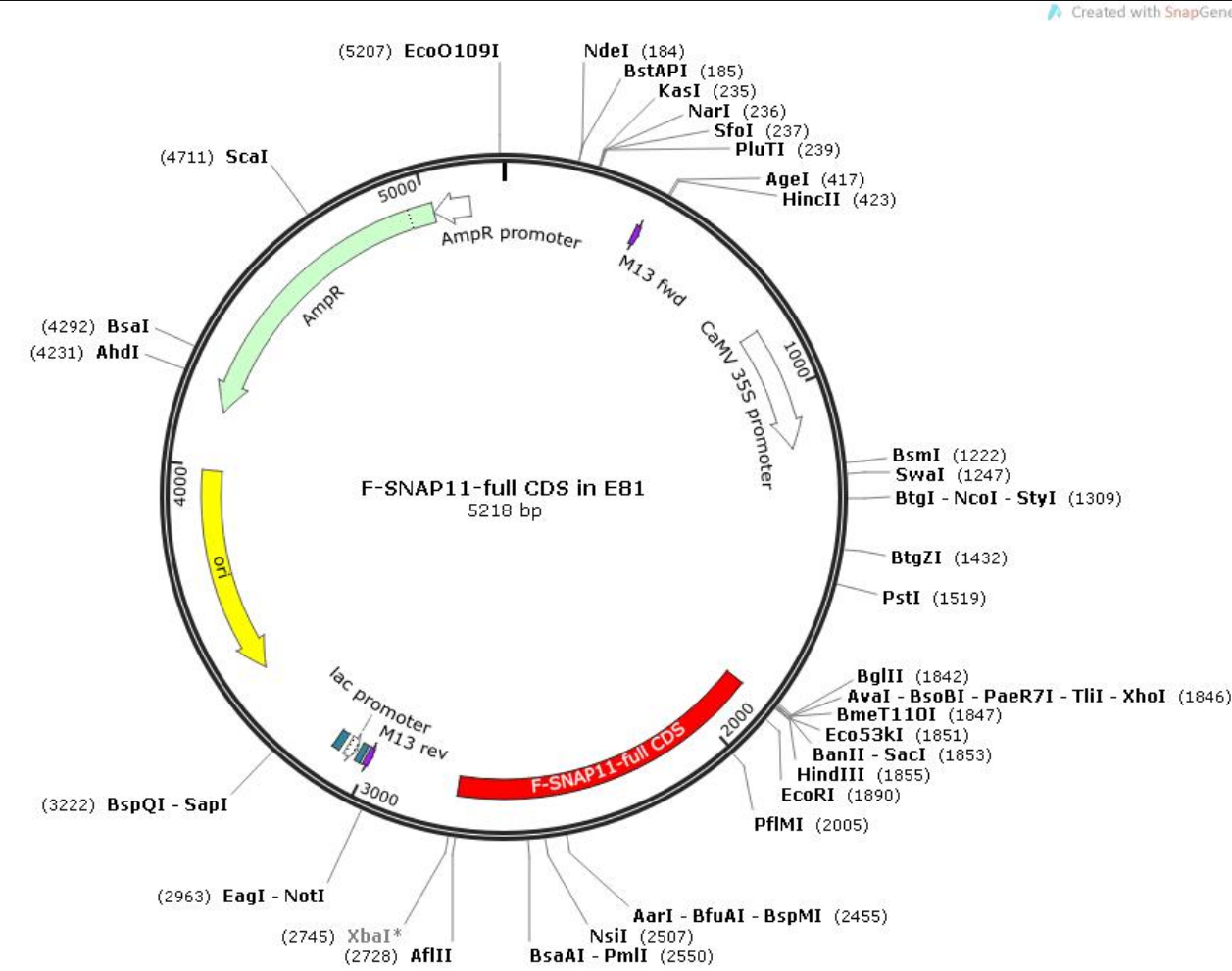

Detailed Sequence of the Whole Construct:

1 TCGCGCGTTT CGGTGATGAC GGTGAAAACC TCTGACACAT GCAGCTCCCG GAGACGGTCA
61 CAGCTTGTCT GTAAGCGGAT GCCGGGAGCA GACAAGCCCG TCAGGGCGCG TCAGCGGGTG
121 TTGGCGGGTG TCGGGGCTGG CTTAACTATG CGGCATCAGA GCAGATTGTA CTGAGAGTGC
181 ACCATATGCG GTGTGAAATA CCGCACAGAT GCGTAAGGAG AAAATACCGC ATCAGGCGCC
241 ATTCGCCATT CAGGCTGCGC AACTGTTGGG AAGGGCGATC GGTGCGGGCC TCTTCGCTAT
301 TACGCCAGCT GGCGAAAGGG GGATGTGCTG CAAGGCGATT AAGTTGGGTA ACGCCAGGGT
361 TTTCCCAGTC ACGACGTTGT AAAACGACGG CCAGTGCCAT TACCCTGTTA TCCCTAACCG
421 GTCAACATGT GGAGCACGAC ACACTTGTCT ACTCCAAAAA TATCAAAGAT ACAGTCTCAG
481 AAGACCAAAG GGCAATTGAG ACTTTTCAAC AAAGGGTAAT ATCCGGAAAC CTCCTCGGAT
541 TCCATTGCCC AGCTATCTGT CACTTTATTG TGAAGATAGT GGAAAAGGAA GGTGGCTCCT
601 ACAAATGCCA TCATTGCGAT AAAGGAAAGG CCATCGTTGA AGATGCCTCT GCCGACAGTG
661 GTCCCAAAGA TGGACCCCCA CCCACGAGGA GCATCGTGGA AAAAGAAGAC GTTCCAACCA
721 CGTCTTCAAA GCAAGTGGAT TGATGTGATA ACATGGTGGA GCACGACACA CTTGTCTACT
781 CCAAAAATAT CAAAGATACA GTCTCAGAAG ACCAAAGGGC AATTGAGACT TTTCAACAAA
841 GGGTAATATC CGGAAACCTC CTCGGATTCC ATTGCCCAGC TATCTGTCAC TTTATTGTGA
901 AGATAGTGGA AAAGGAAGGT GGCTCCTACA AATGCCATCA TTGCGATAAA GGAAAGGCCA
961 TCGTTGAAGA TGCCTCTGCC GACAGTGGTC CCAAAGATGG ACCCCCACCC ACGAGGAGCA
1021 TCGTGGAAAA AGAAGACGTT CCAACCACGT CTTCAAAGCA AGTGGATTGA TGTGATATCT
1081 CCACTGACGT AAGGGATGAC GCACAATCCC ACTATCCTTC GCAAGACCCT TCCTCTATAT
1141 AAGGAAGTTC ATTTCATTTG GAGAGGACGT CGAGAGTTCT CAACACAACA TATACAAAAC
1201 AAACGAATCT CAAGCAATCA AGCATTCTAC TTCTATTGCA GCAATTTAAA TCATTTCTTT
1261 TAAAGCAAAA GCAATTTTCT GAAAATTTTC ACCATTTACG AACGATAGCC ATGGTGAGCA
1321 AGGGCGAGGA GCTGTTCACC GGGGTGGTGC CCATCCTGGT CGAGCTGGAC GGCGACGTAA
1381 ACGGCCACAA GTTCAGCGTG TCCGGCGAGG GCGAGGGCGA TGCCACCTAC GGCAAGCTGA
1441 CCCTGAAGTT CATCTGCACC ACCGGCAAGC TGCCCGTGCC CTGGCCCACC CTCGTGACCA
1501 CCTTCGGCTA CGGCCTGCAG TGCTTCGCCC GCTACCCCGA CCACATGAAG CAGCACGACT
1561 TCTTCAAGTC CGCCATGCCC GAAGGCTACG TCCAGGAGCG CACCATCTTC TTCAAGGACG
1621 ACGGCAACTA CAAGACCCGC GCCGAGGTGA AGTTCGAGGG CGACACCCTG GTGAACCGCA
1681 TCGAGCTGAA GGGCATCGAC TTCAAGGAGG ACGGCAACAT CCTGGGGCAC AAGCTGGAGT
1741 ACAACTACAA CAGCCACAAC GTCTATATCA TGGCCGACAA GCAGAAGAAC GGCATCAAGG
1801 TGAACTTCAA GATCCGCCAC AACATCGAGG ACTCCGGACT CAGATCTCGA GCTCAAGCTT
1861 CGATGGCCGA TCAGTTATCC AAAGGAGAGG AATTCGAGAA GAAAGCTGAG AAGAAGCTCA

```
1921 GCGGTTGGGG CTTGTTTGGC TCCAAGTACG AAGATGCCGC CGATCTCTTC GATAAAGCCG
1981 CCAATTGCTT CAAGCTCGCC AAATCATGGG ACAAAGCTGG AGCGACATAC CTGAAGTTGG
2041 CAAGTTGTCA TTTGAAGTTG GAAAGCAAGC ATGAAGCTGC ACAGGCCCAT GTTGATGCTG
2101 CACATAGCTA TAAAAAAACT AATATAAACG AGTCTGTATC TTGCTTAGAC CAAGCCGTAA
2161 ATCTTTTCTG TGACATTGGA AGACTATCTA TGGCTGCTAG ATATTTAAAG GAAATTGCTG
2221 AATTGTATGA GGGTGAACAG AATATTGAGC AGGCTCTTGT TTACTATGAA AAATCAGCTG
2281 ATTTTTTTCA AAATGAAGAA GTGACAACTT CTGCAAACCA ATGCAAACAA AAAGTTGCCC
2341 AGTTTGCTGC TCAGCTAGAA CAATATCAGA AGTCGATTGA GATTTACGAA GAGATAACTC
2401 GCCAATCCCT CAACAATAAT TTGCTGAAGT ATGGAGTTAA AGGACACCTG CTTAATGCTG
2461 GCATCTGCCA ACTCTGTAAA GGGGATGTTA TTGCTGTAAC CAATGCATTA GAACGATATC
2521 AGGAACTGGA TCCAACATTT TCAGGAACAC GTGAATATAG ATTTTTGGCG GACATTGCTG
2581 CTGCAATTGA TGAAGAAGAT GTTGCGAAGT TTACTGATGT TGTCAAGGAA TTTGATAGCA
2641 TGACCCCTCT GGATTCTTGG AAGACCACAC TTCTTTTGAG GGTGAAGGAA AAGCTGAAAG
2701 CCAAAGAACT TGAGGAGGAT GATCTTACTT AAGGATCCAC CTGATCTAGA GTCCGCAAAA
2761 ATCACCAGTC TCTCTCTACA AATCTATCTC TCTCTATTTT TCTCCAGAAT AATGTGTGAG
2821 TAGTTCCCAG ATAAGGGAAT TAGGGTTCTT ATAGGGTTTC GCTCATGTGT TGAGCATATA
2881 AGAAACCCTT AGTATGTATT TGTATTTGTA AAATACTTCT ATCAATAAAA TTTCTAATTC
2941 CTAAAACCAA AATCCAGTGA CGCGGCCGCA TTACCCTGTT ATCCCTAGTA ATCATGGTCA
3001 TAGCTGTTTC CTGTGTGAAA TTGTTATCCG CTCACAATTC CACACAACAT ACGAGCCGGA
3061 AGCATAAAGT GTAAAGCCTG GGGTGCCTAA TGAGTGAGCT AACTCACATT AATTGCGTTG
3121 CGCTCACTGC CCGCTTTCCA GTCGGGAAAC CTGTCGTGCC AGCTGCATTA ATGAATCGGC
3181 CAACGCGCGG GGAGAGGCGG TTTGCGTATT GGGCGCTCTT CCGCTTCCTC GCTCACTGAC
3241 TCGCTGCGCT CGGTCGTTCG GCTGCGGCGA GCGGTATCAG CTCACTCAAA GGCGGTAATA
3301 CGGTTATCCA CAGAATCAGG GGATAACGCA GGAAAGAACA TGTGAGCAAA AGGCCAGCAA
3361 AAGGCCAGGA ACCGTAAAAA GGCCGCGTTG CTGGCGTTTT TCCATAGGCT CCGCCCCCCT
3421 GACGAGCATC ACAAAAATCG ACGCTCAAGT CAGAGGTGGC GAAACCCGAC AGGACTATAA
3481 AGATACCAGG CGTTTCCCCC TGGAAGCTCC CTCGTGCGCT CTCCTGTTCC GACCCTGCCG
3541 CTTACCGGAT ACCTGTCCGC CTTTCTCCCT TCGGGAAGCG TGGCGCTTTC TCAATGCTCA
3601 CGCTGTAGGT ATCTCAGTTC GGTGTAGGTC GTTCGCTCCA AGCTGGGCTG TGTGCACGAA
3661 CCCCCCGTTC AGCCCGACCG CTGCGCCTTA TCCGGTAACT ATCGTCTTGA GTCCAACCCG
3721 GTAAGACACG ACTTATCGCC ACTGGCAGCA GCCACTGGTA ACAGGATTAG CAGAGCGAGG
3781 TATGTAGGCG GTGCTACAGA GTTCTTGAAG TGGTGGCCTA ACTACGGCTA CACTAGAAGG
3841 ACAGTATTTG GTATCTGCGC TCTGCTGAAG CCAGTTACCT TCGGAAAAAG AGTTGGTAGC
3901 TCTTGATCCG GCAAACAAAC CACCGCTGGT AGCGGTGGTT TTTTTGTTTG CAAGCAGCAG
3961 ATTACGCGCA GAAAAAAAGG ATCTCAAGAA GATCCTTTGA TCTTTTCTAC GGGGTCTGAC
4021 GCTCAGTGGA ACGAAAACTC ACGTTAAGGG ATTTTGGTCA TGAGATTATC AAAAAGGATC
4081 TTCACCTAGA TCCTTTTAAA TTAAAAATGA AGTTTTAAAT CAATCTAAAG TATATATGAG
4141 TAAACTTGGT CTGACAGTTA CCAATGCTTA ATCAGTGAGG CACCTATCTC AGCGATCTGT
4201 CTATTTCGTT CATCCATAGT TGCCTGACTC CCCGTCGTGT AGATAACTAC GATACGGGAG
4261 GGCTTACCAT CTGGCCCCAG TGCTGCAATG ATACCGCGAG ACCCACGCTC ACCGGCTCCA
4321 GATTTATCAG CAATAAACCA GCCAGCCGGA AGGGCCGAGC GCAGAAGTGG TCCTGCAACT
4381 TTATCCGCCT CCATCCAGTC TATTAATTGT TGCCGGGAAG CTAGAGTAAG TAGTTCGCCA
4441 GTTAATAGTT TGCGCAACGT TGTTGCCATT GCTACAGGCA TCGTGGTGTC ACGCTCGTCG
4501 TTTGGTATGG CTTCATTCAG CTCCGGTTCC CAACGATCAA GGCGAGTTAC ATGATCCCCC
4561 ATGTTGTGCA AAAAAGCGGT TAGCTCCTTC GGTCCTCCGA TCGTTGTCAG AAGTAAGTTG
4621 GCCGCAGTGT TATCACTCAT GGTTATGGCA GCACTGCATA ATTCTCTTAC TGTCATGCCA
4681 TCCGTAAGAT GCTTTTCTGT GACTGGTGAG TACTCAACCA AGTCATTCTG AGAATAGTGT
4741 ATGCGGCGAC CGAGTTGCTC TTGCCCGGCG TCAATACGGG ATAATACCGC GCCACATAGC
4801 AGAACTTTAA AAGTGCTCAT CATTGGAAAA CGTTCTTCGG GGCGAAAACT CTCAAGGATC
4861 TTACCGCTGT TGAGATCCAG TTCGATGTAA CCCACTCGTG CACCCAACTG ATCTTCAGCA
4921 TCTTTTACTT TCACCAGCGT TTCTGGGTGA GCAAAAACAG GAAGGCAAAA TGCCGCAAAA
4981 AAGGGAATAA GGGCGACACG GAAATGTTGA ATACTCATAC TCTTCCTTTT TCAATATTAT
5041 TGAAGCATTT ATCAGGGTTA TTGTCTCATG AGCGGATACA TATTTGAATG TATTTAGAAA
5101 AATAAACAAA TAGGGGTTCC GCGCACATTT CCCCGAAAAG TGCCACCTGA CGTCTAAGAA
5161 ACCATTATTA TCATGACATT AACCTATAAA AATAGGCGTA TCACGAGGCC CTTTCGTC
```
