# Supplementary material for: Dissecting nematode resistance regions in soybean revealed pleiotropic effect of soybean cyst and reniform nematode resistance genes
Source: Plant Genome. 2021 Mar 16;14(2):e20083. doi: 10.1002/tpg2.20083 (PMC12807396; doi:10.1002/tpg2.20083)
Supplement: Supplementary file 3 [file TPG2-14-e20083-s004.pdf]

Gene Name E-SNAP11 CDS
Cloning Vector *pSAT4-nEYFP-C1 (E81)*
Length (bp) 884
Cloning Sites HindIII(AAGCTT)-BamHI(GGATCC)

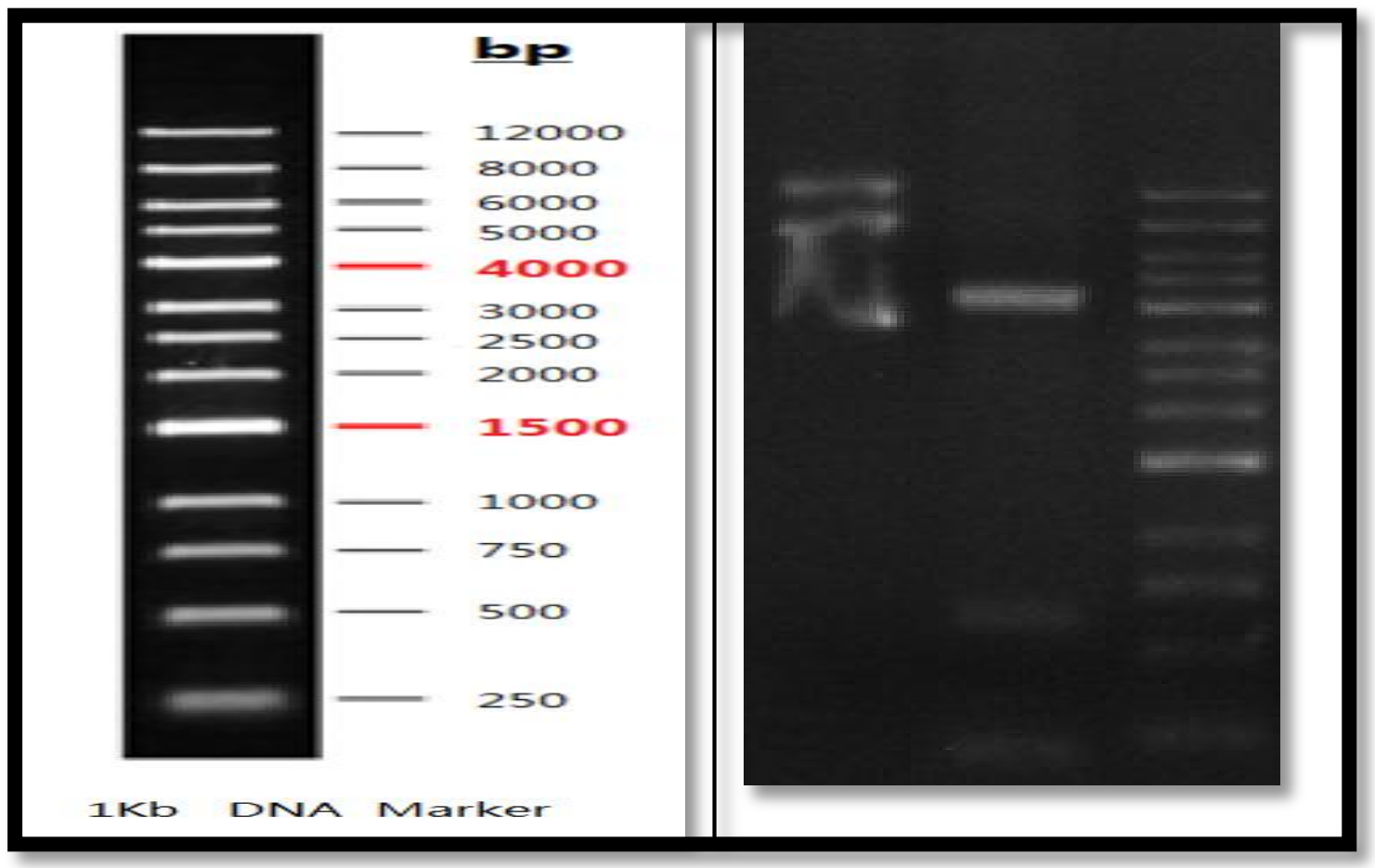

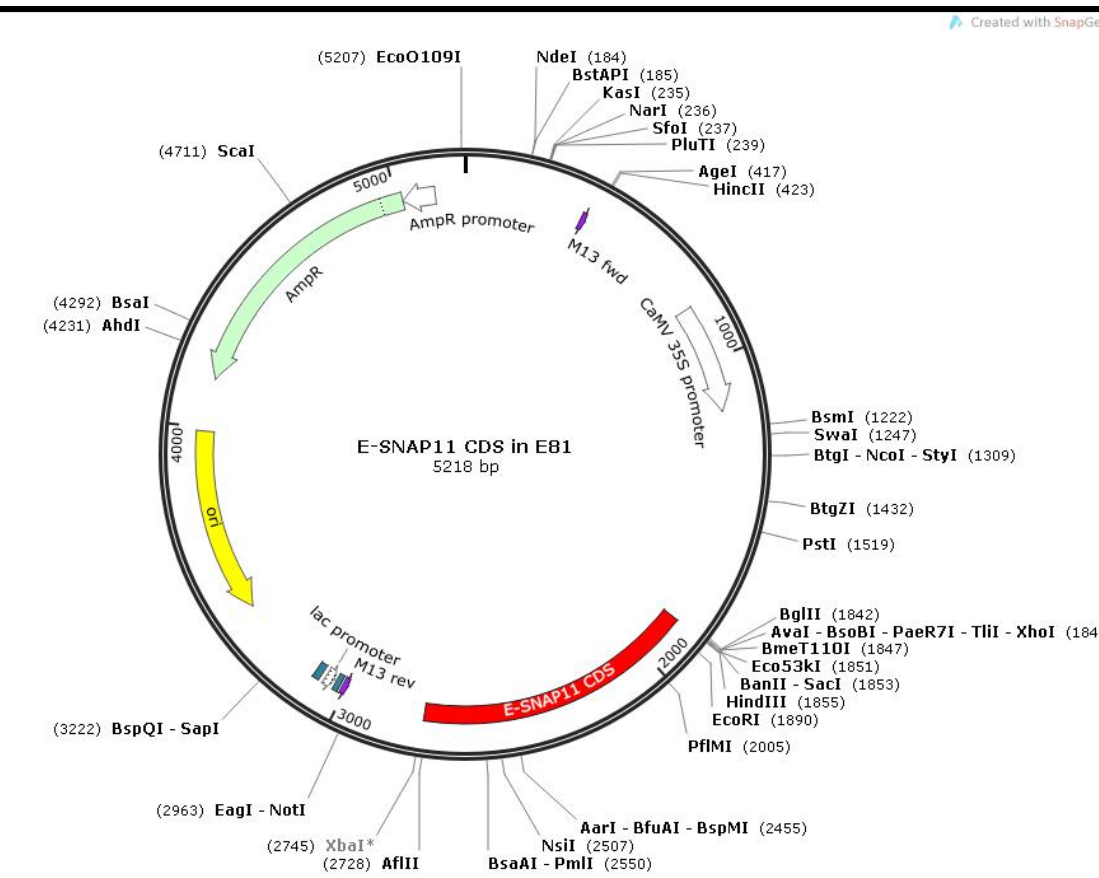

Detailed Sequence of the Whole Construct:

```
1 TCGCGCGTTT CGGTGATGAC GGTGAAAACC TCTGACACAT GCAGCTCCCG GAGACGGTCA
       61 CAGCTTGTCT GTAAGCGGAT GCCGGGAGCA GACAAGCCCG TCAGGGCGCG TCAGCGGGTG
      121 TTGGCGGGTG TCGGGGCTGG CTTAACTATG CGGCATCAGA GCAGATTGTA CTGAGAGTGC
      181 ACCATATGCG GTGTGAAATA CCGCACAGAT GCGTAAGGAG AAAATACCGC ATCAGGCGCC
      241 ATTCGCCATT CAGGCTGCGC AACTGTTGGG AAGGGCGATC GGTGCGGGCC TCTTCGCTAT
      301 TACGCCAGCT GGCGAAAGGG GGATGTGCTG CAAGGCGATT AAGTTGGGTA ACGCCAGGGT
      361 TTTCCCAGTC ACGACGTTGT AAAACGACGG CCAGTGCCAT TACCCTGTTA TCCCTAACCG
      421 GTCAACATGT GGAGCACGAC ACACTTGTCT ACTCCAAAAA TATCAAAGAT ACAGTCTCAG
      481 AAGACCAAAG GGCAATTGAG ACTTTTCAAC AAAGGGTAAT ATCCGGAAAC CTCCTCGGAT
      541 TCCATTGCCC AGCTATCTGT CACTTTATTG TGAAGATAGT GGAAAAGGAA GGTGGCTCCT
      601 ACAAATGCCA TCATTGCGAT AAAGGAAAGG CCATCGTTGA AGATGCCTCT GCCGACAGTG
      661 GTCCCAAAGA TGGACCCCCA CCCACGAGGA GCATCGTGGA AAAAGAAGAC GTTCCAACCA
      721 CGTCTTCAAA GCAAGTGGAT TGATGTGATA ACATGGTGGA GCACGACACA CTTGTCTACT
      781 CCAAAAATAT CAAAGATACA GTCTCAGAAG ACCAAAGGGC AATTGAGACT TTTCAACAAA
      841 GGGTAATATC CGGAAACCTC CTCGGATTCC ATTGCCCAGC TATCTGTCAC TTTATTGTGA
      901 AGATAGTGGA AAAGGAAGGT GGCTCCTACA AATGCCATCA TTGCGATAAA GGAAAGGCCA
      961 TCGTTGAAGA TGCCTCTGCC GACAGTGGTC CCAAAGATGG ACCCCCACCC ACGAGGAGCA
     1021 TCGTGGAAAA AGAAGACGTT CCAACCACGT CTTCAAAGCA AGTGGATTGA TGTGATATCT
     1081 CCACTGACGT AAGGGATGAC GCACAATCCC ACTATCCTTC GCAAGACCCT TCCTCTATAT
     1141 AAGGAAGTTC ATTTCATTTG GAGAGGACGT CGAGAGTTCT CAACACAACA TATACAAAAC
     1201 AAACGAATCT CAAGCAATCA AGCATTCTAC TTCTATTGCA GCAATTTAAA TCATTTCTTT
     1261 TAAAGCAAAA GCAATTTTCT GAAAATTTTC ACCATTTACG AACGATAGCC ATGGTGAGCA
     1321 AGGGCGAGGA GCTGTTCACC GGGGTGGTGC CCATCCTGGT CGAGCTGGAC GGCGACGTAA
     1381 ACGGCCACAA GTTCAGCGTG TCCGGCGAGG GCGAGGGCGA TGCCACCTAC GGCAAGCTGA
     1441 CCCTGAAGTT CATCTGCACC ACCGGCAAGC TGCCCGTGCC CTGGCCCACC CTCGTGACCA
     1501 CCTTCGGCTA CGGCCTGCAG TGCTTCGCCC GCTACCCCGA CCACATGAAG CAGCACGACT
     1561 TCTTCAAGTC CGCCATGCCC GAAGGCTACG TCCAGGAGCG CACCATCTTC TTCAAGGACG
     1621 ACGGCAACTA CAAGACCCGC GCCGAGGTGA AGTTCGAGGG CGACACCCTG GTGAACCGCA
     1681 TCGAGCTGAA GGGCATCGAC TTCAAGGAGG ACGGCAACAT CCTGGGGCAC AAGCTGGAGT
     1741 ACAACTACAA CAGCCACAAC GTCTATATCA TGGCCGACAA GCAGAAGAAC GGCATCAAGG
     1801 TGAACTTCAA GATCCGCCAC AACATCGAGG ACTCCGGACT CAGATCTCGA GCTCAAGCTT
     1861 CGATGGCCGA TCAGTTATCC AAAGGAGAGG AATTCGAGAA GAAAGCTGAG AAGAAGCTCA
     1921 GCGGTTGGGG CTTGTTTGGC TCCAAGTACG AAGATGCCGC CGATCTCTTT GATAAAGCCG
     1981 CCAATTGCTT CAAGCTCGCC AAATCATGGG ACAAAGCTGG AGCGACATAC CTGAAGTTGG
     2041 CAAGTTGTCA TTTGAAGTTG GAAAGCAAGC ATGAAGCTGC ACAGGCCCAT GTTGATGCTG
```

```
2101 CACATAGCTA TAAAAAAACT AATATAAACG AGTCTGTATC TTGCTTAGAC CAAGCCGTAA
2161 ATCTTTTCTG TGACATTGGA AGACTATCTA TGGCTGCTAG ATATTTAAAG GAAATTGCTG
2221 AATTGTATGA GGGTGAACAG AATATTGAGC AGGCTCTTGT TTACTATGAA AAATCAGCTG
2281 ATTTTTTTCA AAATGAAGAA GTGACAACTT CTGCAAACCA ATGCAAACAA AAAGTTGCCC
2341 AGTTTGCTGC TCAGCTAGAA CAATATCAGA AGTCGATTGA GATTTACGAA GAGATAGCTC
2401 GCCAATCCCT CAACAATAAT TTGCTGAAGT ATGGAGTTAA AGGACACCTG CTTAATGCTG
2461 GCATCTGCCA ACTCTGTAAA GGGGATGTTA TTGCTGTAAC CAATGCATTA GAACGATATC
2521 AGGAACTGGA TCCAACATTT TCGGGAACAC GTGAATATAG ATTTTTGGCG GACATTGCTG
2581 CTGCAATTGA TGAAGAAGAT GTTGCGAAGT TTACTGATGT TGTCAAGGAA TTTGATAGCA
2641 TGACCCCTCT GGATTCTTGG AAGACCACAC TTCTTTTGAG GGTGAAGGAA AAGCTGAAAG
2701 CCAAAGAACT TGAGGAGGAT GATCTTACTT AAGGATCCAC CTGATCTAGA GTCCGCAAAA
2761 ATCACCAGTC TCTCTCTACA AATCTATCTC TCTCTATTTT TCTCCAGAAT AATGTGTGAG
2821 TAGTTCCCAG ATAAGGGAAT TAGGGTTCTT ATAGGGTTTC GCTCATGTGT TGAGCATATA
2881 AGAAACCCTT AGTATGTATT TGTATTTGTA AAATACTTCT ATCAATAAAA TTTCTAATTC
2941 CTAAAACCAA AATCCAGTGA CGCGGCCGCA TTACCCTGTT ATCCCTAGTA ATCATGGTCA
3001 TAGCTGTTTC CTGTGTGAAA TTGTTATCCG CTCACAATTC CACACAACAT ACGAGCCGGA
3061 AGCATAAAGT GTAAAGCCTG GGGTGCCTAA TGAGTGAGCT AACTCACATT AATTGCGTTG
3121 CGCTCACTGC CCGCTTTCCA GTCGGGAAAC CTGTCGTGCC AGCTGCATTA ATGAATCGGC
3181 CAACGCGCGG GGAGAGGCGG TTTGCGTATT GGGCGCTCTT CCGCTTCCTC GCTCACTGAC
3241 TCGCTGCGCT CGGTCGTTCG GCTGCGGCGA GCGGTATCAG CTCACTCAAA GGCGGTAATA
3301 CGGTTATCCA CAGAATCAGG GGATAACGCA GGAAAGAACA TGTGAGCAAA AGGCCAGCAA
3361 AAGGCCAGGA ACCGTAAAAA GGCCGCGTTG CTGGCGTTTT TCCATAGGCT CCGCCCCCCT
3421 GACGAGCATC ACAAAAATCG ACGCTCAAGT CAGAGGTGGC GAAACCCGAC AGGACTATAA
3481 AGATACCAGG CGTTTCCCCC TGGAAGCTCC CTCGTGCGCT CTCCTGTTCC GACCCTGCCG
3541 CTTACCGGAT ACCTGTCCGC CTTTCTCCCT TCGGGAAGCG TGGCGCTTTC TCAATGCTCA
3601 CGCTGTAGGT ATCTCAGTTC GGTGTAGGTC GTTCGCTCCA AGCTGGGCTG TGTGCACGAA
3661 CCCCCCGTTC AGCCCGACCG CTGCGCCTTA TCCGGTAACT ATCGTCTTGA GTCCAACCCG
3721 GTAAGACACG ACTTATCGCC ACTGGCAGCA GCCACTGGTA ACAGGATTAG CAGAGCGAGG
3781 TATGTAGGCG GTGCTACAGA GTTCTTGAAG TGGTGGCCTA ACTACGGCTA CACTAGAAGG
3841 ACAGTATTTG GTATCTGCGC TCTGCTGAAG CCAGTTACCT TCGGAAAAAG AGTTGGTAGC
3901 TCTTGATCCG GCAAACAAAC CACCGCTGGT AGCGGTGGTT TTTTTGTTTG CAAGCAGCAG
3961 ATTACGCGCA GAAAAAAAGG ATCTCAAGAA GATCCTTTGA TCTTTTCTAC GGGGTCTGAC
4021 GCTCAGTGGA ACGAAAACTC ACGTTAAGGG ATTTTGGTCA TGAGATTATC AAAAAGGATC
4081 TTCACCTAGA TCCTTTTAAA TTAAAAATGA AGTTTTAAAT CAATCTAAAG TATATATGAG
4141 TAAACTTGGT CTGACAGTTA CCAATGCTTA ATCAGTGAGG CACCTATCTC AGCGATCTGT
4201 CTATTTCGTT CATCCATAGT TGCCTGACTC CCCGTCGTGT AGATAACTAC GATACGGGAG
4261 GGCTTACCAT CTGGCCCCAG TGCTGCAATG ATACCGCGAG ACCCACGCTC ACCGGCTCCA
4321 GATTTATCAG CAATAAACCA GCCAGCCGGA AGGGCCGAGC GCAGAAGTGG TCCTGCAACT
4381 TTATCCGCCT CCATCCAGTC TATTAATTGT TGCCGGGAAG CTAGAGTAAG TAGTTCGCCA
4441 GTTAATAGTT TGCGCAACGT TGTTGCCATT GCTACAGGCA TCGTGGTGTC ACGCTCGTCG
4501 TTTGGTATGG CTTCATTCAG CTCCGGTTCC CAACGATCAA GGCGAGTTAC ATGATCCCCC
4561 ATGTTGTGCA AAAAAGCGGT TAGCTCCTTC GGTCCTCCGA TCGTTGTCAG AAGTAAGTTG
4621 GCCGCAGTGT TATCACTCAT GGTTATGGCA GCACTGCATA ATTCTCTTAC TGTCATGCCA
4681 TCCGTAAGAT GCTTTTCTGT GACTGGTGAG TACTCAACCA AGTCATTCTG AGAATAGTGT
4741 ATGCGGCGAC CGAGTTGCTC TTGCCCGGCG TCAATACGGG ATAATACCGC GCCACATAGC
4801 AGAACTTTAA AAGTGCTCAT CATTGGAAAA CGTTCTTCGG GGCGAAAACT CTCAAGGATC
4861 TTACCGCTGT TGAGATCCAG TTCGATGTAA CCCACTCGTG CACCCAACTG ATCTTCAGCA
4921 TCTTTTACTT TCACCAGCGT TTCTGGGTGA GCAAAAACAG GAAGGCAAAA TGCCGCAAAA
4981 AAGGGAATAA GGGCGACACG GAAATGTTGA ATACTCATAC TCTTCCTTTT TCAATATTAT
5041 TGAAGCATTT ATCAGGGTTA TTGTCTCATG AGCGGATACA TATTTGAATG TATTTAGAAA
5101 AATAAACAAA TAGGGGTTCC GCGCACATTT CCCCGAAAAG TGCCACCTGA CGTCTAAGAA
5161 ACCATTATTA TCATGACATT AACCTATAAA AATAGGCGTA TCACGAGGCC CTTTCGTC
```
