# Supplementary material for: Dissecting nematode resistance regions in soybean revealed pleiotropic effect of soybean cyst and reniform nematode resistance genes
Source: Plant Genome. 2021 Mar 16;14(2):e20083. doi: 10.1002/tpg2.20083 (PMC12807396; doi:10.1002/tpg2.20083)
Supplement: Supplementary file 4 [file TPG2-14-e20083-s005.pdf]

Gene Name E-SNAP18-CDS
Cloning Vector *pSAT4-cEYFP-C1-B (E82)*
Length (bp) 884
Cloning Sites XhoI-XmaI

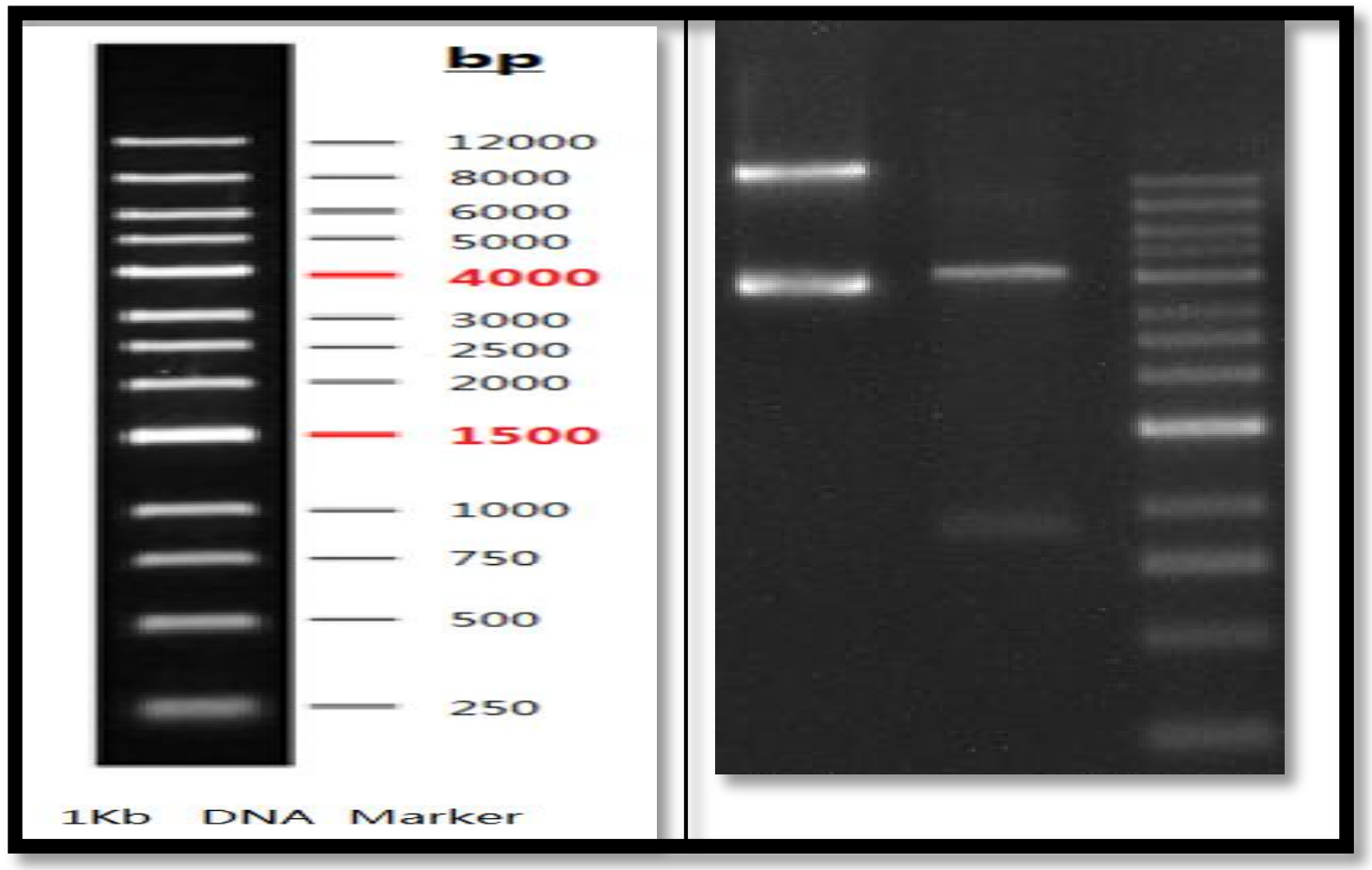

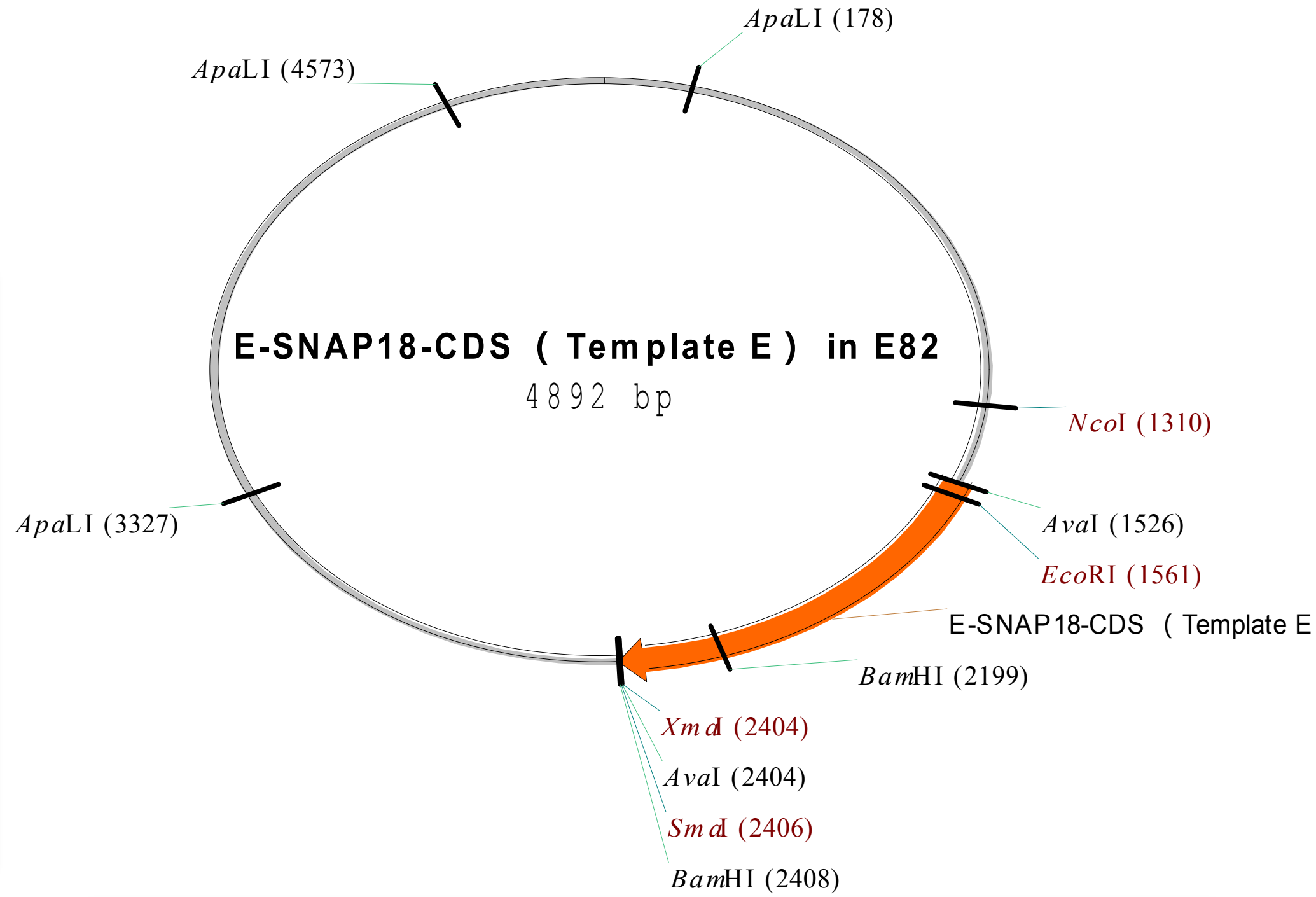

Detailed Sequence of the Whole Construct:

```
   1 TCGCGCGTTT CGGTGATGAC GGTGAAAACC TCTGACACAT GCAGCTCCCG GAGACGGTCA
  61 CAGCTTGTCT GTAAGCGGAT GCCGGGAGCA GACAAGCCCG TCAGGGCGCG TCAGCGGGTG
 121 TTGGCGGGTG TCGGGGCTGG CTTAACTATG CGGCATCAGA GCAGATTGTA CTGAGAGTGC
 181 ACCATATGCG GTGTGAAATA CCGCACAGAT GCGTAAGGAG AAAATACCGC ATCAGGCGCC
 241 ATTCGCCATT CAGGCTGCGC AACTGTTGGG AAGGGCGATC GGTGCGGGCC TCTTCGCTAT
 301 TACGCCAGCT GGCGAAAGGG GGATGTGCTG CAAGGCGATT AAGTTGGGTA ACGCCAGGGT
 361 TTTCCCAGTC ACGACGTTGT AAAACGACGG CCAGTGCCAT TACCCTGTTA TCCCTAACCG
 421 GTCAACATGT GGAGCACGAC ACACTTGTCT ACTCCAAAAA TATCAAAGAT ACAGTCTCAG
 481 AAGACCAAAG GGCAATTGAG ACTTTTCAAC AAAGGGTAAT ATCCGGAAAC CTCCTCGGAT
 541 TCCATTGCCC AGCTATCTGT CACTTTATTG TGAAGATAGT GGAAAAGGAA GGTGGCTCCT
 601 ACAAATGCCA TCATTGCGAT AAAGGAAAGG CCATCGTTGA AGATGCCTCT GCCGACAGTG
 661 GTCCCAAAGA TGGACCCCCA CCCACGAGGA GCATCGTGGA AAAAGAAGAC GTTCCAACCA
 721 CGTCTTCAAA GCAAGTGGAT TGATGTGATA ACATGGTGGA GCACGACACA CTTGTCTACT
 781 CCAAAAATAT CAAAGATACA GTCTCAGAAG ACCAAAGGGC AATTGAGACT TTTCAACAAA
 841 GGGTAATATC CGGAAACCTC CTCGGATTCC ATTGCCCAGC TATCTGTCAC TTTATTGTGA
 901 AGATAGTGGA AAAGGAAGGT GGCTCCTACA AATGCCATCA TTGCGATAAA GGAAAGGCCA
 961 TCGTTGAAGA TGCCTCTGCC GACAGTGGTC CCAAAGATGG ACCCCCACCC ACGAGGAGCA
1021 TCGTGGAAAA AGAAGACGTT CCAACCACGT CTTCAAAGCA AGTGGATTGA TGTGATATCT
1081 CCACTGACGT AAGGGATGAC GCACAATCCC ACTATCCTTC GCAAGACCCT TCCTCTATAT
1141 AAGGAAGTTC ATTTCATTTG GAGAGGACGT CGAGAGTTCT CAACACAACA TATACAAAAC
1201 AAACGAATCT CAAGCAATCA AGCATTCTAC TTCTATTGCA GCAATTTAAA TCATTTCTTT
1261 TAAAGCAAAA GCAATTTTCT GAAAATTTTC ACCATTTACG AACGATAGCC ATGGGCGGCA
1321 GCGTGCAGCT CGCCGACCAC TACCAGCAGA ACACCCCCAT CGGCGACGGC CCCGTGCTGC
1381 TGCCCGACAA CCACTACCTG AGCTACCAGT CCGCCCTGAG CAAAGACCCC AACGAGAAGC
1441 GCGATCACAT GGTCCTGCTG GAGTTCGTGA CCGCCGCCGG GATCACTCTC GGCATGGACG
1501 AGCTGTACAA GTCCGGACTC AGATCTCGAG CTATGGCCGA TCAGTTATCG AAGGGAGAGG
1561 AATTCGAGAA AAAGGCTGAG AAGAAGCTCA GCGGTTGGGG CTTGTTTGGC TCCAAGTATG
1621 AAGATGCCGC CGATCTCTTC GATAAAGCCG CCAATTGCTT CAAGCTCGCC AAATCATGGG
1681 ACAAGGCTGG AGCGACATAC CTGAAGTTGG CAAGTTGTCA TTTGAAGTTG GAAAGCAAGC
1741 ATGAAGCTGC ACAGGCCCAT GTCGATGCTG CACATTGCTA CAAAAAGACT AATATAAACG
1801 AGTCTGTATC TTGCTTAGAC CGAGCTGTAA ATCTTTTCTG TGACATTGGA AGACTCTCTA
```

```
1861 TGGCTGCTAG ATATTTAAAG GAAATTGCTG AATTGTACGA GGGTGAACAG AATATTGAGC
1921 AGGCTCTTGT TTACTATGAA AAATCAGCTG ATTTTTTTCA AAATGAAGAA GTGACAACTT
1981 CTGCGAACCA ATGCAAACAA AAAGTTGCCC AGTTTGCTGC TCAGCTAGAA CAATATCAGA
2041 AGTCGATTGA CATTTATGAA GAGATAGCTC GCCAATCCCT CAACAATAAT TTGCTGAAGT
2101 ATGGAGTTAA AGGACACCTT CTTAATGCTG GCATCTGCCA ACTCTGTAAA GAGGACGTTG
2161 TTGCTATAAC CAATGCATTA GAACGATATC AGGAACTGGA TCCAACATTT TCAGGAACAC
2221 GTGAATATAG ATTGTTGGCG GACATTGCTG CTGCAATTGA TGAAGAAGAT GTTGCAAAGT
2281 TTACTGATGT TGTCAAGGAA TTTGATAGTA TGACCCCTCT GGATTCTTGG AAGACCACAC
2341 TTCTCTTAAG GGTGAAGGAA AAGCTGAAAG CCAAAGAACT TGAGGAGGAT GATCTTACTT
2401 GACCCGGGAT CCACCTGATC TAGAGTCCGC AAAAATCACC AGTCTCTCTC TACAAATCTA
2461 TCTCTCTCTA TTTTTCTCCA GAATAATGTG TGAGTAGTTC CCAGATAAGG GAATTAGGGT
2521 TCTTATAGGG TTTCGCTCAT GTGTTGAGCA TATAAGAAAC CCTTAGTATG TATTTGTATT
2581 TGTAAAATAC TTCTATCAAT AAAATTTCTA ATTCCTAAAA CCAAAATCCA GTGACGCGGC
2641 CGCATTACCC TGTTATCCCT AGTAATCATG GTCATAGCTG TTTCCTGTGT GAAATTGTTA
2701 TCCGCTCACA ATTCCACACA ACATACGAGC CGGAAGCATA AAGTGTAAAG CCTGGGGTGC
2761 CTAATGAGTG AGCTAACTCA CATTAATTGC GTTGCGCTCA CTGCCCGCTT TCCAGTCGGG
2821 AAACCTGTCG TGCCAGCTGC ATTAATGAAT CGGCCAACGC GCGGGGAGAG GCGGTTTGCG
2881 TATTGGGCGC TCTTCCGCTT CCTCGCTCAC TGACTCGCTG CGCTCGGTCG TTCGGCTGCG
2941 GCGAGCGGTA TCAGCTCACT CAAAGGCGGT AATACGGTTA TCCACAGAAT CAGGGGATAA
3001 CGCAGGAAAG AACATGTGAG CAAAAGGCCA GCAAAAGGCC AGGAACCGTA AAAAGGCCGC
3061 GTTGCTGGCG TTTTTCCATA GGCTCCGCCC CCCTGACGAG CATCACAAAA ATCGACGCTC
3121 AAGTCAGAGG TGGCGAAACC CGACAGGACT ATAAAGATAC CAGGCGTTTC CCCCTGGAAG
3181 CTCCCTCGTG CGCTCTCCTG TTCCGACCCT GCCGCTTACC GGATACCTGT CCGCCTTTCT
3241 CCCTTCGGGA AGCGTGGCGC TTTCTCAATG CTCACGCTGT AGGTATCTCA GTTCGGTGTA
3301 GGTCGTTCGC TCCAAGCTGG GCTGTGTGCA CGAACCCCCC GTTCAGCCCG ACCGCTGCGC
3361 CTTATCCGGT AACTATCGTC TTGAGTCCAA CCCGGTAAGA CACGACTTAT CGCCACTGGC
3421 AGCAGCCACT GGTAACAGGA TTAGCAGAGC GAGGTATGTA GGCGGTGCTA CAGAGTTCTT
3481 GAAGTGGTGG CCTAACTACG GCTACACTAG AAGGACAGTA TTTGGTATCT GCGCTCTGCT
3541 GAAGCCAGTT ACCTTCGGAA AAAGAGTTGG TAGCTCTTGA TCCGGCAAAC AAACCACCGC
3601 TGGTAGCGGT GGTTTTTTTG TTTGCAAGCA GCAGATTACG CGCAGAAAAA AAGGATCTCA
3661 AGAAGATCCT TTGATCTTTT CTACGGGGTC TGACGCTCAG TGGAACGAAA ACTCACGTTA
3721 AGGGATTTTG GTCATGAGAT TATCAAAAAG GATCTTCACC TAGATCCTTT TAAATTAAAA
3781 ATGAAGTTTT AAATCAATCT AAAGTATATA TGAGTAAACT TGGTCTGACA GTTACCAATG
3841 CTTAATCAGT GAGGCACCTA TCTCAGCGAT CTGTCTATTT CGTTCATCCA TAGTTGCCTG
3901 ACTCCCCGTC GTGTAGATAA CTACGATACG GGAGGGCTTA CCATCTGGCC CCAGTGCTGC
3961 AATGATACCG CGAGACCCAC GCTCACCGGC TCCAGATTTA TCAGCAATAA ACCAGCCAGC
4021 CGGAAGGGCC GAGCGCAGAA GTGGTCCTGC AACTTTATCC GCCTCCATCC AGTCTATTAA
4081 TTGTTGCCGG GAAGCTAGAG TAAGTAGTTC GCCAGTTAAT AGTTTGCGCA ACGTTGTTGC
4141 CATTGCTACA GGCATCGTGG TGTCACGCTC GTCGTTTGGT ATGGCTTCAT TCAGCTCCGG
4201 TTCCCAACGA TCAAGGCGAG TTACATGATC CCCCATGTTG TGCAAAAAAG CGGTTAGCTC
4261 CTTCGGTCCT CCGATCGTTG TCAGAAGTAA GTTGGCCGCA GTGTTATCAC TCATGGTTAT
4321 GGCAGCACTG CATAATTCTC TTACTGTCAT GCCATCCGTA AGATGCTTTT CTGTGACTGG
4381 TGAGTACTCA ACCAAGTCAT TCTGAGAATA GTGTATGCGG CGACCGAGTT GCTCTTGCCC
4441 GGCGTCAATA CGGGATAATA CCGCGCCACA TAGCAGAACT TTAAAAGTGC TCATCATTGG
4501 AAAACGTTCT TCGGGGCGAA AACTCTCAAG GATCTTACCG CTGTTGAGAT CCAGTTCGAT
4561 GTAACCCACT CGTGCACCCA ACTGATCTTC AGCATCTTTT ACTTTCACCA GCGTTTCTGG
4621 GTGAGCAAAA ACAGGAAGGC AAAATGCCGC AAAAAAGGGA ATAAGGGCGA CACGGAAATG
4681 TTGAATACTC ATACTCTTCC TTTTTCAATA TTATTGAAGC ATTTATCAGG GTTATTGTCT
4741 CATGAGCGGA TACATATTTG AATGTATTTA GAAAAATAAA CAAATAGGGG TTCCGCGCAC
4801 ATTTCCCCGA AAAGTGCCAC CTGACGTCTA AGAAACCATT ATTATCATGA CATTAACCTA
4861 TAAAAATAGG CGTATCACGA GGCCCTTTCG TC
```
