# Supplementary material for: Dissecting nematode resistance regions in soybean revealed pleiotropic effect of soybean cyst and reniform nematode resistance genes
Source: Plant Genome. 2021 Mar 16;14(2):e20083. doi: 10.1002/tpg2.20083 (PMC12807396; doi:10.1002/tpg2.20083)
Supplement: Supplementary file 5 [file TPG2-14-e20083-s008.pdf]

Gene Name *GmSNAP11*$_{A240*}$ CDS
Cloning Vector *pSAT4-nEYFP-C1 (E81)*
Length (bp) 734
Cloning Sites HindIII(AAGCTT)-BamHI(GGATCC)

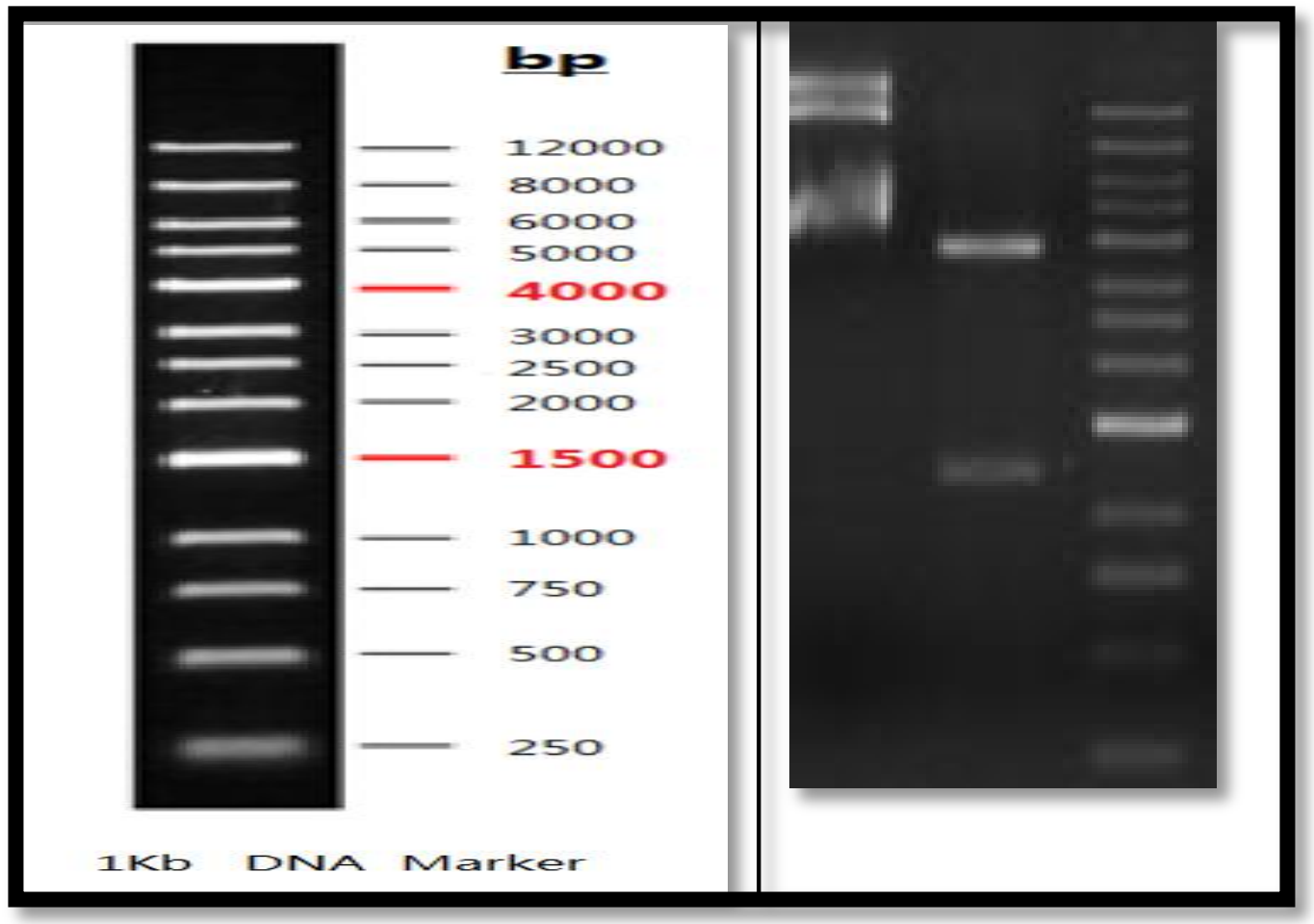

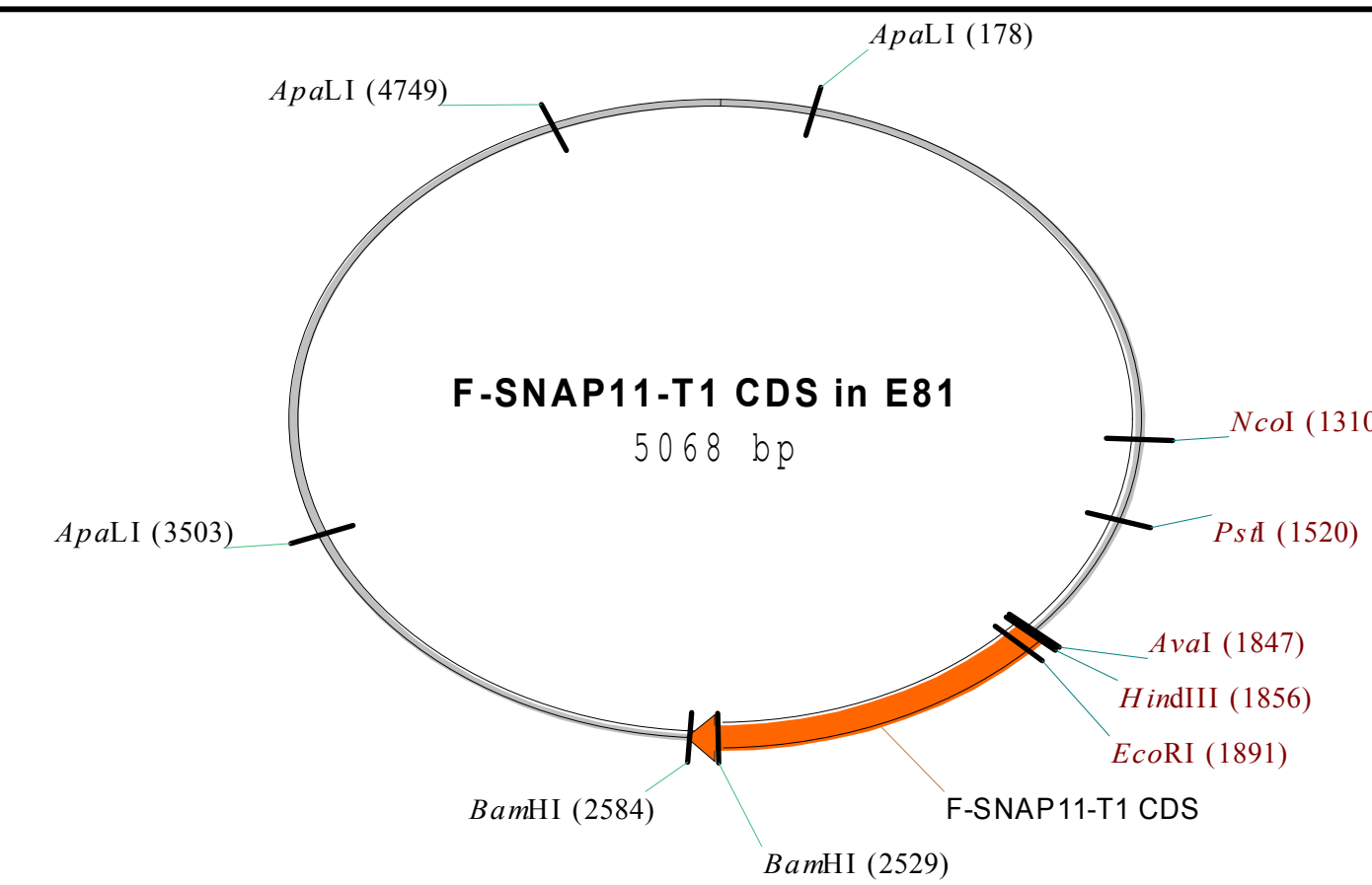

Detailed Sequence of the Whole Construct:

```
   1 TCGCGCGTTT CGGTGATGAC GGTGAAAACC TCTGACACAT GCAGCTCCCG GAGACGGTCA
  61 CAGCTTGTCT GTAAGCGGAT GCCGGGAGCA GACAAGCCCG TCAGGGCGCG TCAGCGGGTG
 121 TTGGCGGGTG TCGGGGCTGG CTTAACTATG CGGCATCAGA GCAGATTGTA CTGAGAGTGC
 181 ACCATATGCG GTGTGAAATA CCGCACAGAT GCGTAAGGAG AAAATACCGC ATCAGGCGCC
 241 ATTCGCCATT CAGGCTGCGC AACTGTTGGG AAGGGCGATC GGTGCGGGCC TCTTCGCTAT
 301 TACGCCAGCT GGCGAAAGGG GGATGTGCTG CAAGGCGATT AAGTTGGGTA ACGCCAGGGT
 361 TTTCCCAGTC ACGACGTTGT AAAACGACGG CCAGTGCCAT TACCCTGTTA TCCCTAACCG
 421 GTCAACATGT GGAGCACGAC ACACTTGTCT ACTCCAAAAA TATCAAAGAT ACAGTCTCAG
 481 AAGACCAAAG GGCAATTGAG ACTTTTCAAC AAAGGGTAAT ATCCGGAAAC CTCCTCGGAT
 541 TCCATTGCCC AGCTATCTGT CACTTTATTG TGAAGATAGT GGAAAAGGAA GGTGGCTCCT
 601 ACAAATGCCA TCATTGCGAT AAAGGAAAGG CCATCGTTGA AGATGCCTCT GCCGACAGTG
 661 GTCCCAAAGA TGGACCCCCA CCCACGAGGA GCATCGTGGA AAAAGAAGAC GTTCCAACCA
 721 CGTCTTCAAA GCAAGTGGAT TGATGTGATA ACATGGTGGA GCACGACACA CTTGTCTACT
 781 CCAAAAATAT CAAAGATACA GTCTCAGAAG ACCAAAGGGC AATTGAGACT TTTCAACAAA
 841 GGGTAATATC CGGAAACCTC CTCGGATTCC ATTGCCCAGC TATCTGTCAC TTTATTGTGA
 901 AGATAGTGGA AAAGGAAGGT GGCTCCTACA AATGCCATCA TTGCGATAAA GGAAAGGCCA
 961 TCGTTGAAGA TGCCTCTGCC GACAGTGGTC CCAAAGATGG ACCCCCACCC ACGAGGAGCA
1021 TCGTGGAAAA AGAAGACGTT CCAACCACGT CTTCAAAGCA AGTGGATTGA TGTGATATCT
1081 CCACTGACGT AAGGGATGAC GCACAATCCC ACTATCCTTC GCAAGACCCT TCCTCTATAT
1141 AAGGAAGTTC ATTTCATTTG GAGAGGACGT CGAGAGTTCT CAACACAACA TATACAAAAC
1201 AAACGAATCT CAAGCAATCA AGCATTCTAC TTCTATTGCA GCAATTTAAA TCATTTCTTT
1261 TAAAGCAAAA GCAATTTTCT GAAAATTTTC ACCATTTACG AACGATAGCC ATGGTGAGCA
1321 AGGGCGAGGA GCTGTTCACC GGGGTGGTGC CCATCCTGGT CGAGCTGGAC GGCGACGTAA
1381 ACGGCCACAA GTTCAGCGTG TCCGGCGAGG GCGAGGGCGA TGCCACCTAC GGCAAGCTGA
1441 CCCTGAAGTT CATCTGCACC ACCGGCAAGC TGCCCGTGCC CTGGCCCACC CTCGTGACCA
1501 CCTTCGGCTA CGGCCTGCAG TGCTTCGCCC GCTACCCCGA CCACATGAAG CAGCACGACT
1561 TCTTCAAGTC CGCCATGCCC GAAGGCTACG TCCAGGAGCG CACCATCTTC TTCAAGGACG
1621 ACGGCAACTA CAAGACCCGC GCCGAGGTGA AGTTCGAGGG CGACACCCTG GTGAACCGCA
1681 TCGAGCTGAA GGGCATCGAC TTCAAGGAGG ACGGCAACAT CCTGGGGCAC AAGCTGGAGT
1741 ACAACTACAA CAGCCACAAC GTCTATATCA TGGCCGACAA GCAGAAGAAC GGCATCAAGG
1801 TGAACTTCAA GATCCGCCAC AACATCGAGG ACTCCGGACT CAGATCTCGA GCTCAAGCTT
1861 CGATGGCCGA TCAGTTATCC AAAGGAGAGG AATTCGAGAA GAAAGCTGAG AAGAAGCTCA
1921 GCGGTTGGGG CTTGTTTGGC TCCAAGTACG AAGATGCCGC CGATCTCTTC GATAAAGCCG
```

```
1981 CCAATTGCTT CAAGCTCGCC AAATCATGGG ACAAAGCTGG AGCGACATAC CTGAAGTTGG
2041 CAAGTTGTCA TTTGAAGTTG GAAAGCAAGC ATGAAGCTGC ACAGGCCCAT GTTGATGCTG
2101 CACATAGCTA TAAAAAAACT AATATAAACG AGTCTGTATC TTGCTTAGAC CAAGCCGTAA
2161 ATCTTTTCTG TGACATTGGA AGACTATCTA TGGCTGCTAG ATATTTAAAG GAAATTGCTG
2221 AATTGTATGA GGGTGAACAG AATATTGAGC AGGCTCTTGT TTACTATGAA AAATCAGCTG
2281 ATTTTTTTCA AAATGAAGAA GTGACAACTT CTGCAAACCA ATGCAAACAA AAAGTTGCCC
2341 AGTTTGCTGC TCAGCTAGAA CAATATCAGA AGTCGATTGA GATTTACGAA GAGATAACTC
2401 GCCAATCCCT CAACAATAAT TTGCTGAAGT ATGGAGTTAA AGGACACCTG CTTAATGCTG
2461 GCATCTGCCA ACTCTGTAAA GGGGATGTTA TTGCTGTAAC CAATGCATTA GAACGATATC
2521 AGGAACTGGA TCCAACATTT TCAGGAACAC GTGAATATAG ATTTTTGGCG TTAGGTCACT
2581 AGGGATCCAC CTGATCTAGA GTCCGCAAAA ATCACCAGTC TCTCTCTACA AATCTATCTC
2641 TCTCTATTTT TCTCCAGAAT AATGTGTGAG TAGTTCCCAG ATAAGGGAAT TAGGGTTCTT
2701 ATAGGGTTTC GCTCATGTGT TGAGCATATA AGAAACCCTT AGTATGTATT TGTATTTGTA
2761 AAATACTTCT ATCAATAAAA TTTCTAATTC CTAAAACCAA AATCCAGTGA CGCGGCCGCA
2821 TTACCCTGTT ATCCCTAGTA ATCATGGTCA TAGCTGTTTC CTGTGTGAAA TTGTTATCCG
2881 CTCACAATTC CACACAACAT ACGAGCCGGA AGCATAAAGT GTAAAGCCTG GGGTGCCTAA
2941 TGAGTGAGCT AACTCACATT AATTGCGTTG CGCTCACTGC CCGCTTTCCA GTCGGGAAAC
3001 CTGTCGTGCC AGCTGCATTA ATGAATCGGC CAACGCGCGG GGAGAGGCGG TTTGCGTATT
3061 GGGCGCTCTT CCGCTTCCTC GCTCACTGAC TCGCTGCGCT CGGTCGTTCG GCTGCGGCGA
3121 GCGGTATCAG CTCACTCAAA GGCGGTAATA CGGTTATCCA CAGAATCAGG GGATAACGCA
3181 GGAAAGAACA TGTGAGCAAA AGGCCAGCAA AAGGCCAGGA ACCGTAAAAA GGCCGCGTTG
3241 CTGGCGTTTT TCCATAGGCT CCGCCCCCCT GACGAGCATC ACAAAAATCG ACGCTCAAGT
3301 CAGAGGTGGC GAAACCCGAC AGGACTATAA AGATACCAGG CGTTTCCCCC TGGAAGCTCC
3361 CTCGTGCGCT CTCCTGTTCC GACCCTGCCG CTTACCGGAT ACCTGTCCGC CTTTCTCCCT
3421 TCGGGAAGCG TGGCGCTTTC TCAATGCTCA CGCTGTAGGT ATCTCAGTTC GGTGTAGGTC
3481 GTTCGCTCCA AGCTGGGCTG TGTGCACGAA CCCCCCGTTC AGCCCGACCG CTGCGCCTTA
3541 TCCGGTAACT ATCGTCTTGA GTCCAACCCG GTAAGACACG ACTTATCGCC ACTGGCAGCA
3601 GCCACTGGTA ACAGGATTAG CAGAGCGAGG TATGTAGGCG GTGCTACAGA GTTCTTGAAG
3661 TGGTGGCCTA ACTACGGCTA CACTAGAAGG ACAGTATTTG GTATCTGCGC TCTGCTGAAG
3721 CCAGTTACCT TCGGAAAAAG AGTTGGTAGC TCTTGATCCG GCAAACAAAC CACCGCTGGT
3781 AGCGGTGGTT TTTTTGTTTG CAAGCAGCAG ATTACGCGCA GAAAAAAAGG ATCTCAAGAA
3841 GATCCTTTGA TCTTTTCTAC GGGGTCTGAC GCTCAGTGGA ACGAAAACTC ACGTTAAGGG
3901 ATTTTGGTCA TGAGATTATC AAAAAGGATC TTCACCTAGA TCCTTTTAAA TTAAAAATGA
3961 AGTTTTAAAT CAATCTAAAG TATATATGAG TAAACTTGGT CTGACAGTTA CCAATGCTTA
4021 ATCAGTGAGG CACCTATCTC AGCGATCTGT CTATTTCGTT CATCCATAGT TGCCTGACTC
4081 CCCGTCGTGT AGATAACTAC GATACGGGAG GGCTTACCAT CTGGCCCCAG TGCTGCAATG
4141 ATACCGCGAG ACCCACGCTC ACCGGCTCCA GATTTATCAG CAATAAACCA GCCAGCCGGA
4201 AGGGCCGAGC GCAGAAGTGG TCCTGCAACT TTATCCGCCT CCATCCAGTC TATTAATTGT
4261 TGCCGGGAAG CTAGAGTAAG TAGTTCGCCA GTTAATAGTT TGCGCAACGT TGTTGCCATT
4321 GCTACAGGCA TCGTGGTGTC ACGCTCGTCG TTTGGTATGG CTTCATTCAG CTCCGGTTCC
4381 CAACGATCAA GGCGAGTTAC ATGATCCCCC ATGTTGTGCA AAAAAGCGGT TAGCTCCTTC
4441 GGTCCTCCGA TCGTTGTCAG AAGTAAGTTG GCCGCAGTGT TATCACTCAT GGTTATGGCA
4501 GCACTGCATA ATTCTCTTAC TGTCATGCCA TCCGTAAGAT GCTTTTCTGT GACTGGTGAG
4561 TACTCAACCA AGTCATTCTG AGAATAGTGT ATGCGGCGAC CGAGTTGCTC TTGCCCGGCG
4621 TCAATACGGG ATAATACCGC GCCACATAGC AGAACTTTAA AAGTGCTCAT CATTGGAAAA
4681 CGTTCTTCGG GGCGAAAACT CTCAAGGATC TTACCGCTGT TGAGATCCAG TTCGATGTAA
4741 CCCACTCGTG CACCCAACTG ATCTTCAGCA TCTTTTACTT TCACCAGCGT TTCTGGGTGA
4801 GCAAAAACAG GAAGGCAAAA TGCCGCAAAA AAGGGAATAA GGGCGACACG GAAATGTTGA
4861 ATACTCATAC TCTTCCTTTT TCAATATTAT TGAAGCATTT ATCAGGGTTA TTGTCTCATG
4921 AGCGGATACA TATTTGAATG TATTTAGAAA AATAAACAAA TAGGGGTTCC GCGCACATTT
4981 CCCCGAAAAG TGCCACCTGA CGTCTAAGAA ACCATTATTA TCATGACATT AACCTATAAA
5041 AATAGGCGTA TCACGAGGCC CTTTCGTC
```
