# Supplementary material for: Dissecting nematode resistance regions in soybean revealed pleiotropic effect of soybean cyst and reniform nematode resistance genes
Source: Plant Genome. 2021 Mar 16;14(2):e20083. doi: 10.1002/tpg2.20083 (PMC12807396; doi:10.1002/tpg2.20083)
Supplement: Supplementary file 6 [file TPG2-14-e20083-s012.pdf]

Gene Name F-SNAP18-CDS
Cloning Vector *pSAT4-cEYFP-C1-B (E82)*
Length (bp) 887
Cloning Sites XhoI-XmaI

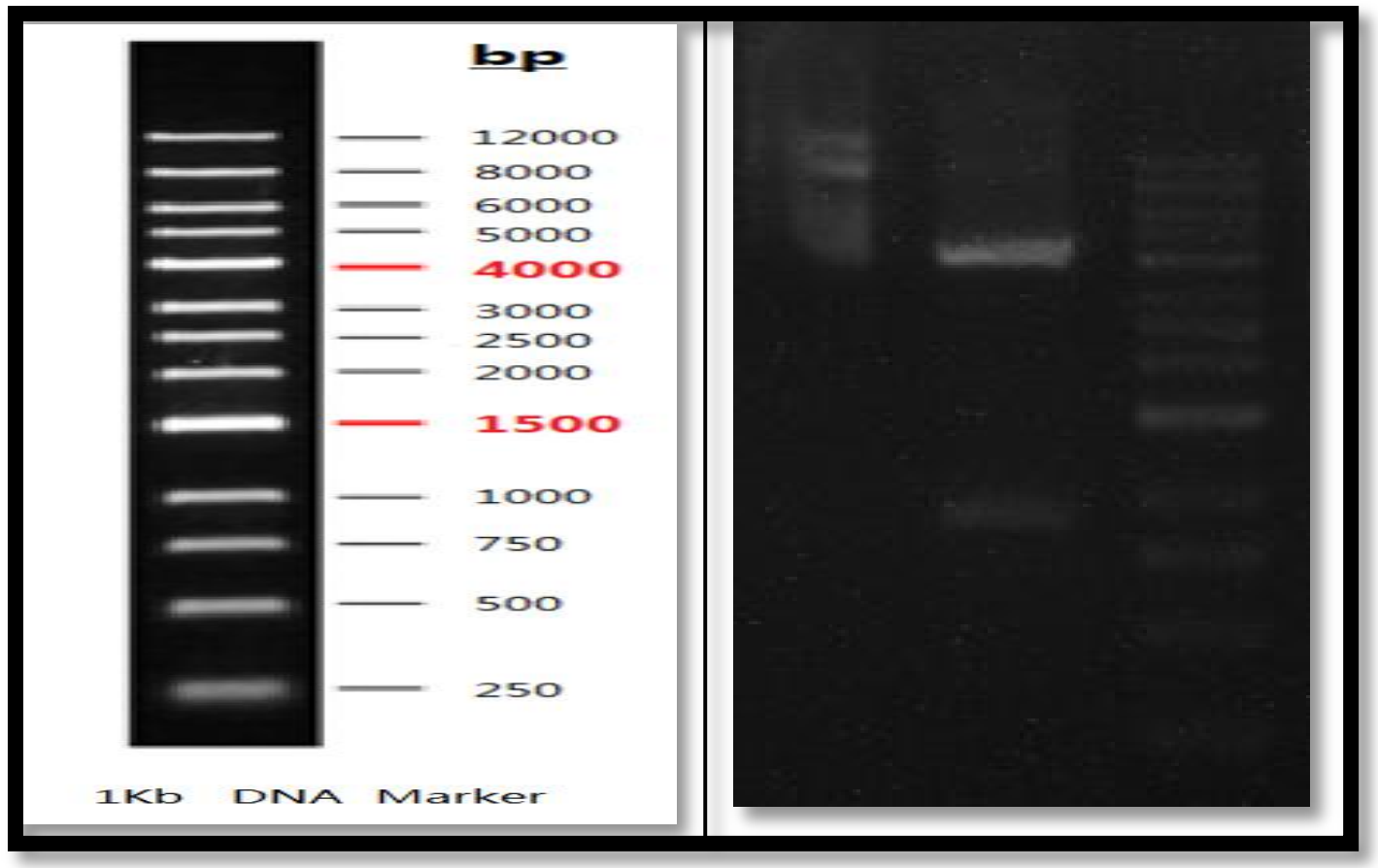

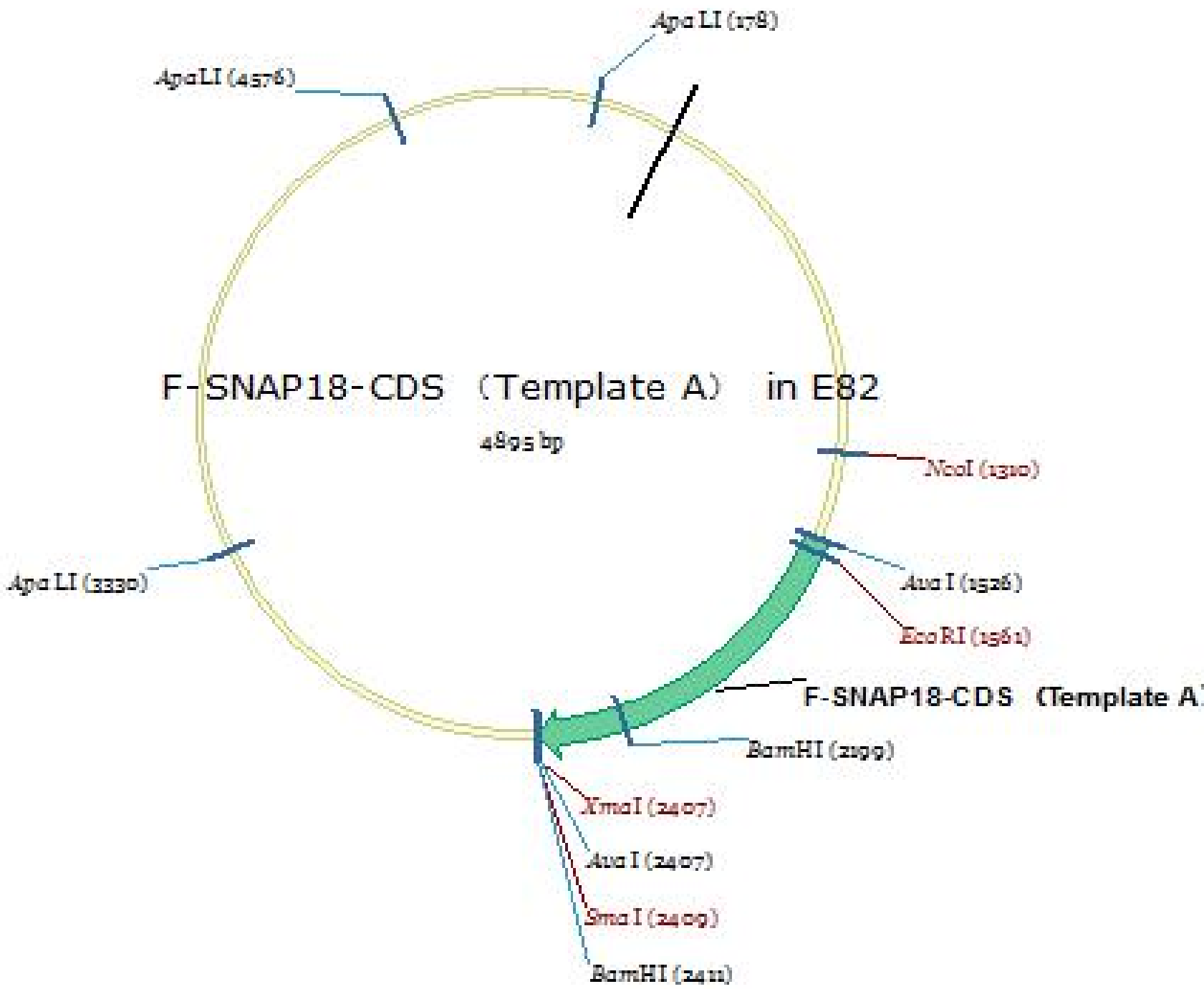

Detailed Sequence of the Whole Construct:

```
   1 TCGCGCGTTT CGGTGATGAC GGTGAAAACC TCTGACACAT GCAGCTCCCG GAGACGGTCA
  61 CAGCTTGTCT GTAAGCGGAT GCCGGGAGCA GACAAGCCCG TCAGGGCGCG TCAGCGGGTG
 121 TTGGCGGGTG TCGGGGCTGG CTTAACTATG CGGCATCAGA GCAGATTGTA CTGAGAGTGC
 181 ACCATATGCG GTGTGAAATA CCGCACAGAT GCGTAAGGAG AAAATACCGC ATCAGGCGCC
 241 ATTCGCCATT CAGGCTGCGC AACTGTTGGG AAGGGCGATC GGTGCGGGCC TCTTCGCTAT
 301 TACGCCAGCT GGCGAAAGGG GGATGTGCTG CAAGGCGATT AAGTTGGGTA ACGCCAGGGT
 361 TTTCCCAGTC ACGACGTTGT AAAACGACGG CCAGTGCCAT TACCCTGTTA TCCCTAACCG
 421 GTCAACATGT GGAGCACGAC ACACTTGTCT ACTCCAAAAA TATCAAAGAT ACAGTCTCAG
 481 AAGACCAAAG GGCAATTGAG ACTTTTCAAC AAAGGGTAAT ATCCGGAAAC CTCCTCGGAT
 541 TCCATTGCCC AGCTATCTGT CACTTTATTG TGAAGATAGT GGAAAAGGAA GGTGGCTCCT
 601 ACAAATGCCA TCATTGCGAT AAAGGAAAGG CCATCGTTGA AGATGCCTCT GCCGACAGTG
 661 GTCCCAAAGA TGGACCCCCA CCCACGAGGA GCATCGTGGA AAAAGAAGAC GTTCCAACCA
 721 CGTCTTCAAA GCAAGTGGAT TGATGTGATA ACATGGTGGA GCACGACACA CTTGTCTACT
 781 CCAAAAATAT CAAAGATACA GTCTCAGAAG ACCAAAGGGC AATTGAGACT TTTCAACAAA
 841 GGGTAATATC CGGAAACCTC CTCGGATTCC ATTGCCCAGC TATCTGTCAC TTTATTGTGA
 901 AGATAGTGGA AAAGGAAGGT GGCTCCTACA AATGCCATCA TTGCGATAAA GGAAAGGCCA
 961 TCGTTGAAGA TGCCTCTGCC GACAGTGGTC CCAAAGATGG ACCCCCACCC ACGAGGAGCA
1021 TCGTGGAAAA AGAAGACGTT CCAACCACGT CTTCAAAGCA AGTGGATTGA TGTGATATCT
1081 CCACTGACGT AAGGGATGAC GCACAATCCC ACTATCCTTC GCAAGACCCT TCCTCTATAT
1141 AAGGAAGTTC ATTTCATTTG GAGAGGACGT CGAGAGTTCT CAACACAACA TATACAAAAC
1201 AAACGAATCT CAAGCAATCA AGCATTCTAC TTCTATTGCA GCAATTTAAA TCATTTCTTT
1261 TAAAGCAAAA GCAATTTTCT GAAAATTTTC ACCATTTACG AACGATAGCC ATGGGCGGCA
1321 GCGTGCAGCT CGCCGACCAC TACCAGCAGA ACACCCCCAT CGGCGACGGC CCCGTGCTGC
1381 TGCCCGACAA CCACTACCTG AGCTACCAGT CCGCCCTGAG CAAAGACCCC AACGAGAAGC
1441 GCGATCACAT GGTCCTGCTG GAGTTCGTGA CCGCCGCCGG GATCACTCTC GGCATGGACG
1501 AGCTGTACAA GTCCGGACTC AGATCTCGAG CTATGGCCGA TCAGTTATCG AAGGGAGAGG
1561 AATTCGAGAA AAAGGCTGAG AAGAAGCTCA GCGGTTGGGG CTTGTTTGGC TCCAAGTATG
```

```
1621 AAGATGCCGC CGATCTCTTC GATAAAGCCG CCAATTGCTT CAAGCTCGCC AAATCATGGG
1681 ACAAGGCTGG AGCGACATAC CTGAAGTTGG CAAGTTGTCA TTTGAAGTTG GAAAGCAAGC
1741 ATGAAGCTGC ACAGGCCCAT GTCGATGCTG CACATTGCTA CAAAAAGACT AATATAAACG
1801 AGTCTGTATC TTGCTTAGAC CGAGCTGTAA ATCTTTTCTG TGACATTGGA AGACTCTCTA
1861 TGGCTGCTAG ATATTTAAAG GAAATTGCTG AATTGTACGA GGGTGAACAG AATATTGAGC
1921 AGGCTCTTGT TTACTATGAA AAATCAGCTG ATTTTTTTCA AAATGAAGAA GTGACAACTT
1981 CTGCGAACCA ATGCAAACAA AAAGTTGCCC AGTTTGCTGC TCAGCTAGAA CAATATCAGA
2041 AGTCGATTGA CATTTATGAA GAGATAGCTC GCCAATCCCT CAACAATAAT TTGCTGAAGT
2101 ATGGAGTTAA AGGACACCTT CTTAATGCTG GCATCTGCCA ACTCTGTAAA GAGGAGGTTG
2161 TTGCTATAAC CAATGCATTA GAACGATATC AGGAACTGGA TCCAACATTT TCAGGAACAC
2221 GTGAATATAG ATTGTTGGCG GACATTGCTG CTGCAATTGA TGAAGAAGAT GTTGCAAAGT
2281 TTACTGATGT TGTCAAGGAA TTTGATAGTA TGACCCCTCT GGATTCTTGG AAGACCACAC
2341 TTCTCTTAAG GGTGAAGGAA AAGCTGAAAG CCAAAGAACT TGAGGAGTAT GAGGTTATTA
2401 CTTGACCCGG GATCCACCTG ATCTAGAGTC CGCAAAAATC ACCAGTCTCT CTCTACAAAT
2461 CTATCTCTCT CTATTTTTCT CCAGAATAAT GTGTGAGTAG TTCCCAGATA AGGGAATTAG
2521 GGTTCTTATA GGGTTTCGCT CATGTGTTGA GCATATAAGA AACCCTTAGT ATGTATTTGT
2581 ATTTGTAAAA TACTTCTATC AATAAAATTT CTAATTCCTA AAACCAAAAT CCAGTGACGC
2641 GGCCGCATTA CCCTGTTATC CCTAGTAATC ATGGTCATAG CTGTTTCCTG TGTGAAATTG
2701 TTATCCGCTC ACAATTCCAC ACAACATACG AGCCGGAAGC ATAAAGTGTA AAGCCTGGGG
2761 TGCCTAATGA GTGAGCTAAC TCACATTAAT TGCGTTGCGC TCACTGCCCG CTTTCCAGTC
2821 GGGAAACCTG TCGTGCCAGC TGCATTAATG AATCGGCCAA CGCGCGGGGA GAGGCGGTTT
2881 GCGTATTGGG CGCTCTTCCG CTTCCTCGCT CACTGACTCG CTGCGCTCGG TCGTTCGGCT
2941 GCGGCGAGCG GTATCAGCTC ACTCAAAGGC GGTAATACGG TTATCCACAG AATCAGGGGA
3001 TAACGCAGGA AAGAACATGT GAGCAAAAGG CCAGCAAAAG GCCAGGAACC GTAAAAAGGC
3061 CGCGTTGCTG GCGTTTTTCC ATAGGCTCCG CCCCCCTGAC GAGCATCACA AAAATCGACG
3121 CTCAAGTCAG AGGTGGCGAA ACCCGACAGG ACTATAAAGA TACCAGGCGT TTCCCCCTGG
3181 AAGCTCCCTC GTGCGCTCTC CTGTTCCGAC CCTGCCGCTT ACCGGATACC TGTCCGCCTT
3241 TCTCCCTTCG GGAAGCGTGG CGCTTTCTCA ATGCTCACGC TGTAGGTATC TCAGTTCGGT
3301 GTAGGTCGTT CGCTCCAAGC TGGGCTGTGT GCACGAACCC CCCGTTCAGC CCGACCGCTG
3361 CGCCTTATCC GGTAACTATC GTCTTGAGTC CAACCCGGTA AGACACGACT TATCGCCACT
3421 GGCAGCAGCC ACTGGTAACA GGATTAGCAG AGCGAGGTAT GTAGGCGGTG CTACAGAGTT
3481 CTTGAAGTGG TGGCCTAACT ACGGCTACAC TAGAAGGACA GTATTTGGTA TCTGCGCTCT
3541 GCTGAAGCCA GTTACCTTCG GAAAAAGAGT TGGTAGCTCT TGATCCGGCA AACAAACCAC
3601 CGCTGGTAGC GGTGGTTTTT TTGTTTGCAA GCAGCAGATT ACGCGCAGAA AAAAAGGATC
3661 TCAAGAAGAT CCTTTGATCT TTTCTACGGG GTCTGACGCT CAGTGGAACG AAAACTCACG
3721 TTAAGGGATT TTGGTCATGA GATTATCAAA AAGGATCTTC ACCTAGATCC TTTTAAATTA
3781 AAAATGAAGT TTTAAATCAA TCTAAAGTAT ATATGAGTAA ACTTGGTCTG ACAGTTACCA
3841 ATGCTTAATC AGTGAGGCAC CTATCTCAGC GATCTGTCTA TTTCGTTCAT CCATAGTTGC
3901 CTGACTCCCC GTCGTGTAGA TAACTACGAT ACGGGAGGGC TTACCATCTG GCCCCAGTGC
3961 TGCAATGATA CCGCGAGACC CACGCTCACC GGCTCCAGAT TTATCAGCAA TAAACCAGCC
4021 AGCCGGAAGG GCCGAGCGCA GAAGTGGTCC TGCAACTTTA TCCGCCTCCA TCCAGTCTAT
4081 TAATTGTTGC CGGGAAGCTA GAGTAAGTAG TTCGCCAGTT AATAGTTTGC GCAACGTTGT
4141 TGCCATTGCT ACAGGCATCG TGGTGTCACG CTCGTCGTTT GGTATGGCTT CATTCAGCTC
4201 CGGTTCCCAA CGATCAAGGC GAGTTACATG ATCCCCCATG TTGTGCAAAA AAGCGGTTAG
4261 CTCCTTCGGT CCTCCGATCG TTGTCAGAAG TAAGTTGGCC GCAGTGTTAT CACTCATGGT
4321 TATGGCAGCA CTGCATAATT CTCTTACTGT CATGCCATCC GTAAGATGCT TTTCTGTGAC
4381 TGGTGAGTAC TCAACCAAGT CATTCTGAGA ATAGTGTATG CGGCGACCGA GTTGCTCTTG
4441 CCCGGCGTCA ATACGGGATA ATACCGCGCC ACATAGCAGA ACTTTAAAAG TGCTCATCAT
4501 TGGAAAACGT TCTTCGGGGC GAAAACTCTC AAGGATCTTA CCGCTGTTGA GATCCAGTTC
4561 GATGTAACCC ACTCGTGCAC CCAACTGATC TTCAGCATCT TTTACTTTCA CCAGCGTTTC
4621 TGGGTGAGCA AAAACAGGAA GGCAAAATGC CGCAAAAAAG GGAATAAGGG CGACACGGAA
4681 ATGTTGAATA CTCATACTCT TCCTTTTTCA ATATTATTGA AGCATTTATC AGGGTTATTG
4741 TCTCATGAGC GGATACATAT TTGAATGTAT TTAGAAAAAT AAACAAATAG GGGTTCCGCG
4801 CACATTTCCC CGAAAAGTGC CACCTGACGT CTAAGAAACC ATTATTATCA TGACATTAAC
4861 CTATAAAAAT AGGCGTATCA CGAGGCCCTT TCGTC
```
