# Supplementary material for: Dissecting nematode resistance regions in soybean revealed pleiotropic effect of soybean cyst and reniform nematode resistance genes
Source: Plant Genome. 2021 Mar 16;14(2):e20083. doi: 10.1002/tpg2.20083 (PMC12807396; doi:10.1002/tpg2.20083)
Supplement: Supplementary file 7 [file TPG2-14-e20083-s003.docx]

**Table S2.** Comparison of physical position of *GmSNAP18* gene at the *rhg1* locus (orange) with RN resistance QTL on Chr.18 (green) reported in this and previous studies.

| **QTL/Locus** | **Accession** | **Confidence Interval / Peak** | **Physical Position (Glyma2.0)** | **Interval** | **LOD** | ***R^2^*** | **Reference** |
| --- | --- | --- | --- | --- | --- | --- | --- |
| ***GmSNAP18*** | . | a-SNAP Gene = Glyma.18g022500 | Gm18:1641008-1645711 | . | . | . | Cook et al., 2012 |
|  |  |  |  |  |  |  |  |
| **RN QTL Chr.18** | PI 437654 | Sat_168 | near Gm18:01706305 | . | 4.1 | 8.0 | Ha et al. 2007 |
|  | PI 567516C | BARC-042717-08388–BARC-048275-10534 | Gm18:01232181-01705500 | 473 kb | 3.5 | 7.5 | Jiao et al. 2015 |
|  | Hartwig | Satt275-Satt163-Satt309 | Gm18:01240120-01736779 | 497 kb | . | . | Lee et al. 2016 |
|  | PI 438489B | BARC-055551-13421 - BARC-048275-10534 | Gm18:01308798 - 01705500 | 397 kb | 4.1 | 7.7 | THIS STUDY |
|  | PI 438489B | bin013 – bin014 | Gm18:01502699 - 01778708 | 276 kb | 4.4 | 7.3 | THIS STUDY |
