# Supplementary material for: Dissecting nematode resistance regions in soybean revealed pleiotropic effect of soybean cyst and reniform nematode resistance genes
Source: Plant Genome. 2021 Mar 16;14(2):e20083. doi: 10.1002/tpg2.20083 (PMC12807396; doi:10.1002/tpg2.20083)
Supplement: Supplementary file 8 [file TPG2-14-e20083-s006.docx]

**Table S3.** Estimation of CNV of *GmSNAP18* and *GmSNAP11* using comparative genome hybridization (CGH) and whole genome re-sequence (WGRS) in NAM population. The data was accessed from the Stupar Lab, the University of Minnesota, MN (http://stuparlabcnv.cfans.umn.edu:8080/).

| **Parent Name** | **Type** |  | ***GmSNAP11*** | |  | ***GmSNAP18*** | |
| --- | --- | --- | --- | --- | --- | --- | --- |
|  |  |  | **CGH** | **WGRS** |  | **CGH** | **WGRS** |
| IA3023 | Parent |  | 1.03 | 0.69 |  | 1.03 | 0.81 |
| TN05-3027 | High Yield |  | 0.94 | 0.75 |  | 1.01 | 0.79 |
| 4J105-3-4 | High Yield |  | 1.41 | 0.70 |  | 5.97 | 8.47 |
| 5M20-2-5-2 | High Yield |  | 0.93 | 0.80 |  | 1.01 | 0.80 |
| CL0J095-4-6 | High Yield |  | 1.42 | 0.72 |  | 5.86 | 7.98 |
| CL0J173-6-8 | High Yield |  | 1.04 | 0.81 |  | 1.03 | 0.88 |
| HS6-3976 | High Yield |  | 1.05 | 0.84 |  | 1.02 | 0.88 |
| Prohio | High Yield |  | 1.08 | 0.63 |  | 1.08 | 0.71 |
| LD00-3309 | High Yield |  | 1.37 | 0.72 |  | 6.42 | 8.15 |
| LD01-5907 | High Yield |  | 1.03 | 0.77 |  | 2.19 | 2.33 |
| LD02-4485 | High Yield |  | 1.41 | 0.73 |  | 6.79 | 7.86 |
| LD02-9050 | High Yield |  | 1.35 | 0.61 |  | 6.58 | 7.95 |
| Magellan | High Yield |  | 1.05 | 0.59 |  | 0.99 | 0.84 |
| Maverick | High Yield |  | 1.27 | 0.74 |  | 6.40 | 8.78 |
| S06-13640 | High Yield |  | 0.96 | 0.53 |  | 1.02 | 0.80 |
| NE3001 | High Yield |  | 1.06 | 0.68 |  | 0.98 | 0.89 |
| Skylla | High Yield |  | 1.13 | 0.77 |  | 1.06 | 0.69 |
| U03-100612 | High Yield |  | 1.03 | 0.72 |  | 1.02 | 0.76 |
| LG03-2979 | Diverse Ancestry |  | 1.06 | 0.74 |  | 1.07 | 0.84 |
| LG03-3191 | Diverse Ancestry |  | 1.03 | 0.62 |  | 1.07 | 0.94 |
| LG04-4717 | Diverse Ancestry |  | 1.03 | 0.74 |  | 1.09 | 0.84 |
| LG05-4292 | Diverse Ancestry |  | 1.21 | 0.76 |  | 5.54 | 7.22 |
| LG05-4317 | Diverse Ancestry |  | 1.15 | 0.73 |  | 0.96 | 0.87 |
| LG05-4464 | Diverse Ancestry |  | 1.07 | 0.76 |  | 1.07 | 0.79 |
| LG05-4832 | Diverse Ancestry |  | 1.06 | 0.65 |  | 1.05 | 0.77 |
| LG90-2550 | Diverse Ancestry |  | 1.04 | 0.72 |  | 1.09 | 0.99 |
| LG92-1255 | Diverse Ancestry |  | 1.02 | 0.79 |  | 1.03 | 0.84 |
| LG94-1128 | Diverse Ancestry |  | 1.06 | 0.75 |  | 1.01 | 1.09 |
| LG94-1906 | Diverse Ancestry |  | 1.05 | 0.73 |  | 1.06 | 0.78 |
| LG97-7012 | Diverse Ancestry |  | 1.06 | 0.66 |  | 1.06 | 0.84 |
| LG98-1605 | Diverse Ancestry |  | 1.03 | 0.78 |  | 1.04 | 0.78 |
| LG00-3372 | Diverse Ancestry |  | 1.03 | 0.55 |  | 1.04 | 0.78 |
| LG04-6000 | Diverse Ancestry |  | 1.04 | 0.83 |  | 0.93 | 1.01 |
| PI 398881 | High Yield + Drought |  | 1.06 | 0.78 |  | 1.07 | 1.02 |
| PI 427136 | High Yield + Drought |  | 1.04 | 0.73 |  | 1.01 | 0.86 |
| PI 437169B | High Yield + Drought |  | 1.04 | 0.75 |  | 0.93 | 0.79 |
| PI 507681B | High Yield + Drought |  | 1.06 | 0.70 |  | 1.05 | 0.77 |
| PI 518751 | High Yield + Drought |  | 1.03 | 0.75 |  | 1.05 | 1.03 |
| PI 561370 | High Yield + Drought |  | 1.03 | 0.86 |  | 1.04 | 0.76 |
| PI 404188A | High Yield + Drought |  | 1.01 | 0.86 |  | 1.04 | 0.86 |
| PI 574486 | High Yield + Drought |  | 1.02 | 0.80 |  | 1.05 | 0.86 |
