# Supplementary material for: Dissecting nematode resistance regions in soybean revealed pleiotropic effect of soybean cyst and reniform nematode resistance genes
Source: Plant Genome. 2021 Mar 16;14(2):e20083. doi: 10.1002/tpg2.20083 (PMC12807396; doi:10.1002/tpg2.20083)
Supplement: Supplementary file 9 [file TPG2-14-e20083-s009.docx]

**Table S4.** Primer sequences for diagnostic SNP assays detecting *Rotylenchulus reniformis* (RN) resistance.

|  | **Primer** | **Position** | **PI88/Pek/W82** | **Forward_FAM** | **Forward_HEX** | **Reverse_Common** |
| --- | --- | --- | --- | --- | --- | --- |
| 1 | SNAP11-1 | Gm11_32968127 | T/T/A | AAGATGAACAACTAGACAGACACACAT | AAGATGAACAACTAGACAGACACACAA | GGCGGTTGGACCTATTGTTTGATGAT |
| 2 | SNAP11-2 | Gm11_32970557 | G/G/T | GGCCTTTATTTTGTTCAGTACCCTTT | GGCCTTTATTTTGTTCAGTACCCTTC | CTAATGTTAACCTGAGGAGAGTGGGTT |
| 3 | SNAP18-1 | Gm18_1645012 | G/G/T | CCTTAGTATTAATATTATACTGAAAAACTAAAG | CCTTAGTATTAATATTATACTGAAAAACTAAAT | TTTTTACACTAACACCCCTAATAAGTGTCA |
| 4 | SNAP18-2 | Gm18_1643107 | G/G/C | CAACCTACACTGACCTAAGCTAATC | CAACCTACACTGACCTAAGCTAATG | CTCTAACCCACTCTCCTCAGGTTAA |
