# Supplementary material for: Dissecting nematode resistance regions in soybean revealed pleiotropic effect of soybean cyst and reniform nematode resistance genes
Source: Plant Genome. 2021 Mar 16;14(2):e20083. doi: 10.1002/tpg2.20083 (PMC12807396; doi:10.1002/tpg2.20083)
Supplement: Supplementary file 10 [file TPG2-14-e20083-s010.docx]

**Table S1.** Comparison of physical position of *GmSNAP11* gene (orange) with RN resistance QTL on Chr.11 (green) and SCN resistance *qSCN11* (blue) reported in this and previous studies.

| **QTL/Locus** | **Race** | **Accession** | **Confidence Interval / Peak** | **Physical Position (Glyma2.0)** | **Interval** | **LOD** | **R2** | **Reference** |
| --- | --- | --- | --- | --- | --- | --- | --- | --- |
| ***GmSNAP11*** | . | . | a-SNAP Gene = Glyma.11g234500 | Gm11:32967943-32972705 |  |  |  | Lakhssassi et al. 2017 |
|  |  |  |  |  |  |  |  |  |
| **RN QTL Chr.11** | RN | PI 437654 | Satt359-Satt484 | Gm11:32411352-33593871 | 1,182 kb | 8.8 | 16 | Ha et al. 2007 |
|  | RN | PI 567516C | Satt444–BARC-042299-08241 | Gm11:29759358-32433567 | 2,674 kb | 4.2 | 8.9 | Jiao et al. 2015 |
|  | RN | PI 438489B | Sat_123 - BARC-018869-03031 | Gm11:32194583 - 33581636 | 1,387 kb | 4.7 | 11.3 | THIS STUDY |
|  | RN | PI 438489B | bin122 – bin124 | Gm11:32892556 - 32978390 | 85 kb | 5.5 | 9.3 | THIS STUDY |
|  |  |  |  |  |  |  |  |  |
| **SCN *qSCN11*** | SCN-1 | PI 089772 | A006-Satt583 | near Gm11:36660484 | . | 6.8 | 16.6 | Yue et al. 2001a |
|  | SCN-2 | PI 089772 | A006-Satt583 | near Gm11:36660484 | . | 2.8 | 6.8 | Yue et al. 2001a |
|  | SCN-5 | PI 089772 | A118-A006 | near Gm11:36660484 | . | 3.7 | 9.5 | Yue et al. 2001a |
|  | SCN-1 | PI 438489B | Sat_123-Satt583 | Gm11:32194528-36660484 | 4,465 kb | 4.2 | 12.7 | Yue et al. 2001b |
|  | SCN-2 | PI 438489B | Sat_123-Satt583 | Gm11:32194528-36660484 | 4,465 kb | 2.8 | 7.4 | Yue et al. 2001b |
|  | SCN-5 | PI 438489B | Sat_123-Satt583 | Gm11:32194528-36660484 | 4,465 kb | 2.7 | 11 | Yue et al. 2001b |
|  | SCN-2 | PI 090763 | Satt359-Satt453 | Gm11:32411352-34173264 | 1,761 kb | 3 | 6.7 | Guo et al. 2005 |
|  | SCN-5 | PI 090763 | Satt359-Satt453 | Gm11:32411352-34173264 | 1,761 kb | 6 | 11.2 | Guo et al. 2005 |
|  | SCN-2 | PI 404198A | Satt453 | near Gm11:34173264 | . | 5.5 | 11.1 | Guo et al. 2006 |
|  | SCN-5 | PI 404198A | Satt453 | near Gm11:34173264 | . | 6.7 | 13 | Guo et al. 2006 |
|  | SCN-1 | PI 437654 | Satt665-EacaMctt067 | near Gm11:31754126 | . | . | . | Wu et al. 2009 |
|  | SCN-3 | PI 437654 | EacaMctt067-Satt484 | near Gm11:31754126 | . | . | . | Wu et al. 2009 |
|  | SCN-5 | PI 437654 | Satt665-EacaMctt067 | near Gm11:31754126 | . | . | . | Wu et al. 2009 |
